# Human CSF movement influenced by vascular low frequency oscillations and respiration

**DOI:** 10.3389/fphys.2022.940140

**Published:** 2022-08-19

**Authors:** Vidhya Vijayakrishnan Nair, Brianna R. Kish, Ben Inglis, Ho-Ching (Shawn) Yang, Adam M. Wright, Yu-Chien Wu, Xiaopeng Zhou, Amy J. Schwichtenberg, Yunjie Tong

**Affiliations:** ^1^ Weldon School of Biomedical Engineering, Purdue University, West Lafayette, IN, United States; ^2^ Henry H. Wheeler, Jr. Brain Imaging Center, Helen Wills Neuroscience Institute, University of California, Berkeley, Berkeley, CA, United States; ^3^ Department of Radiology and Imaging Sciences, Indiana University School of Medicine, Indianapolis, IN, United States; ^4^ Stark Neuroscience Research Institute, Indiana University School of Medicine, Indianapolis, IN, United States; ^5^ School of Health Sciences, Purdue University, West Lafayette, IN, United States; ^6^ Department of Human Development and Family Studies, College of Health and Human Sciences, Purdue University, West Lafayette, IN, United States

**Keywords:** cerebrospinal fluid, low-frequency oscillations, respiration, neck fMRI scans, hemodynamics

## Abstract

Cerebrospinal fluid (CSF) movement through the pathways within the central nervous system is of high significance for maintaining normal brain health and function. Low frequency hemodynamics and respiration have been shown to drive CSF in humans independently. Here, we hypothesize that CSF movement may be driven simultaneously (and in synchrony) by both mechanisms and study their independent and coupled effects on CSF movement using novel neck fMRI scans. Caudad CSF movement at the fourth ventricle and hemodynamics of the major neck blood vessels (internal carotid arteries and internal jugular veins) was measured from 11 young, healthy volunteers using novel neck fMRI scans with simultaneous measurement of respiration. Two distinct models of CSF movement (1. Low-frequency hemodynamics and 2. Respiration) and possible coupling between them were investigated. We show that the dynamics of brain fluids can be assessed from the neck by studying the interrelationships between major neck blood vessels and the CSF movement in the fourth ventricle. We also demonstrate that there exists a cross-frequency coupling between these two separable mechanisms. The human CSF system can respond to multiple coupled physiological forces at the same time. This information may help inform the pathological mechanisms behind CSF movement-related disorders.

## 1 Introduction

Cerebrospinal Fluid (CSF), an ultrafiltrate of blood plasma, flows through the ventricles of the human brain and the subarachnoid spaces of the cranium and spine. Predominantly secreted by the choroid plexuses, CSF is known to perform several critical functions essential for brain health and function (Sakka, Coll and Chazal, 2011). In addition to offering mechanical protection and support to the brain and spinal cord, it also maintains the brain homeostasis by transporting nutrients, hormones and other immunologic factors through the central nervous system (CNS) (Spector and Johanson, 2007; Schwartz and Baruch, 2012; Spector, Robert Snodgrass and Johanson, 2015).

The flow of CSF in the CNS garnered increased interest after the relatively recent proposal of the glymphatic system. According to this model, the flow of CSF through the brain’s perivascular pathways and further exchange with the interstitial fluid (ISF) is instrumental in removing the metabolic waste products of the brain (Iliff et al., 2013a; Jessen et al., 2015). Furthermore, emerging evidence has also linked dysfunction of CSF pathways to increased amyloid-beta concentrations in the context of neurodegenerative disorders such as Alzheimer’s disease (Ueno et al., 2014; Han et al., 2021) and elevated tau concentrations with chronic traumatic encephalopathy (Puvenna et al., 2016).

Despite the crucial physiological roles and clinical significance of CSF dynamics, the exact driving forces behind CSF flow in humans are still poorly understood. Cardiac pulsations were the first physiological mechanism to be considered as a potential motive force behind CSF flow. Based on this model, the intracranial arterial expansion due to the arrival of cardiac pulsations leads to brain expansion which in turn compresses the brain ventricles resulting in caudally directed CSF flow from the brain into the spinal canal through the brain ventricles (Greitz et al., 1992). Observations of bidirectional pulsatile CSF flow (caudad and cephalad) dependent on the cardiac cycle, at the cervical level, have also been reported using conventional cardiac gated phase contrast Magnetic Resonance Imaging (MRI) based measurements (Enzmann and Pelc, 1993; Bhadelia, Bogdan and Wolpert, 1995). Several animal model studies have also explained a brain-wide perivascular pathway that aids the exchange between CSF and ISF for interstitial waste clearance, primarily driven by cerebral arterial pulsations (Iliff et al., 2013a; Iliff et al., 2013b; Xie et al., 2013). A similar pulsation driven absorption of CSF by the brain capillaries have also been reported in humans (Greitz and Hannerz, 1996; Greitz, 2004). However, recent studies based on mathematical modelling argue that these cardiac pulsations alone are too weak to drive this clearance (Asgari, De Zélicourt and Kurtcuoglu, 2016; Diem et al., 2017). A recent study on awake mice has reported spontaneous contractions and relaxations of the vascular smooth muscle cells in the low frequency range (<0.1 Hz), referred to as vasomotion, to be the principal driver for this perivascular clearance pathway (van Veluw et al., 2020).

Other mechanisms are also currently debated in the literature as the motive force propelling CSF flow in humans. Studies using different forms of phase contrast MRI have identified respiration as a major regulator of CSF flow (Yamada et al., 2013; Chen et al., 2015; Dreha-Kulaczewski et al., 2015, 2017). Specifically, Dreha-Kulaczewski et al., quantified a net upward flow of CSF into the cranium from the spinal canal and its relation to the venous outflow, during a deep breathing regimen. More recently, a functional MRI (fMRI) study by Fultz et al. (2019) reported coupling between cranially directed CSF flow in the fourth ventricle and cerebral hemodynamic signals in the low frequency range (<0.1 Hz) during non-rapid eye movement (NREM) sleep. Expanding on this line of work, we recently proposed a biomechanical model of CSF dynamics, suggesting low frequency brain hemodynamic signals (0.01–0.1 Hz) as the fundamental driving force for CSF movement in both directions (i.e., cranial and caudal), in the awake state (Yang et al., 2021).

In this study, we use fMRI to explore the different mechanisms driving the CSF movement. We hypothesize that more than one mechanism simultaneously (and in synchrony) drives the CSF movement: 1) A hemodynamic mechanism that drives CSF movement in the low-frequency range (0.01–0.1 Hz), and 2) respiration, which explains CSF movement in the respiratory frequency range (0.2–0.4 Hz). For this study, we conducted neck fMRI scans (instead of typical brain scans) for the following reasons: 1) All the brain fluids (blood, CSF) are transported into/out of the brain through the neck so that the flow dynamics in the neck offers a simple, direct way to assess the input/output fluid dynamics of the brain, as well as their relationships; 2) Respiration has been shown to directly and mechanically affect the large draining veins in the neck [i.e., inspiration leading to collapse of the internal jugular veins (IJV)], in turn leading to internal pressure changes in the brain. Therefore, measuring these signals in the neck and their relationships to respiration are crucial. Furthermore, we explore the cross-frequency coupling between respiration and low-frequency oscillations (LFOs), particularly in the context of the observed CSF dynamics.

## 2 Materials and methods

### 2.1 Participants

This study included a total of 11 healthy participants (7 females and 4 males) aged 20–25 (21.4 ± 1.4) years. The study was approved by Purdue University’s Human Research Protection Plan (IRB-2020-1329) and was conducted in accordance with application of Belmont Report principles (Respect for Persons, Beneficence, and Justice) and federal regulations 45 CFR 46, 21 CFR 50, 56. Written informed consent was obtained from all participants.

### 2.2 Mechanisms of cerebrospinal fluid dynamics

The unique feature of the neck fMRI scan (Yang et al., 2021) is used in this study to explore different mechanisms that contribute to CSF dynamics. Here we investigate two distinct models based on the classic Monro-Kellie doctrine which stipulates that the total volume of brain parenchyma, CSF, and intracranial blood remains constant: 1) a hemodynamic model in the low frequency range: 0.01–0.1 Hz and 2) a respiration model in the respiratory frequency range: 0.2–0.4 Hz. The Monroe-Kellie doctrine implies that a change in volume of any one brain component would lead to a compensatory change in volume of at least one of the other components (Mokri, 2001).

#### 2.2.1 Hemodynamic model

For the hemodynamic model, we hypothesize that the net intravascular blood volume changes in the low-frequency range in both the arteries (main contribution) and veins are the dominant forces moving the CSF. As found previously, an intravascular blood volume change initially detected in Internal Carotid Arteries (ICAs) can be detected later in the IJVs (Tong et al., 2018). Since ICAs are the main blood supply to the brain, the arterial volume changes in ICAs precede the cerebral blood volume (CBV) changes in the brain tissue, before eventually producing a net Blood oxygen level dependent (BOLD) signal change in the IJVs. (Note that the ICAs produce a BOLD signal change, but for normal oxygen saturation >98% the arterial blood is more diamagnetic than water and this produces an inverted BOLD signal compared to the later positive BOLD signal in the IJVs.) The cumulative effects of CBV changes within the brain parenchyma (i.e., net CBV) exert forces on the lateral ventricle walls. As a result, the CSF will be forced in/out via the fourth ventricle (Yang et al., 2021). In parallel, the CBV change in arteries and arterioles leads to passive blood volume and flow changes in the draining veins (Supplementary Note S1). The latter evokes detectable BOLD signal in the IJVs a few seconds later.

**FIGURE 1:**
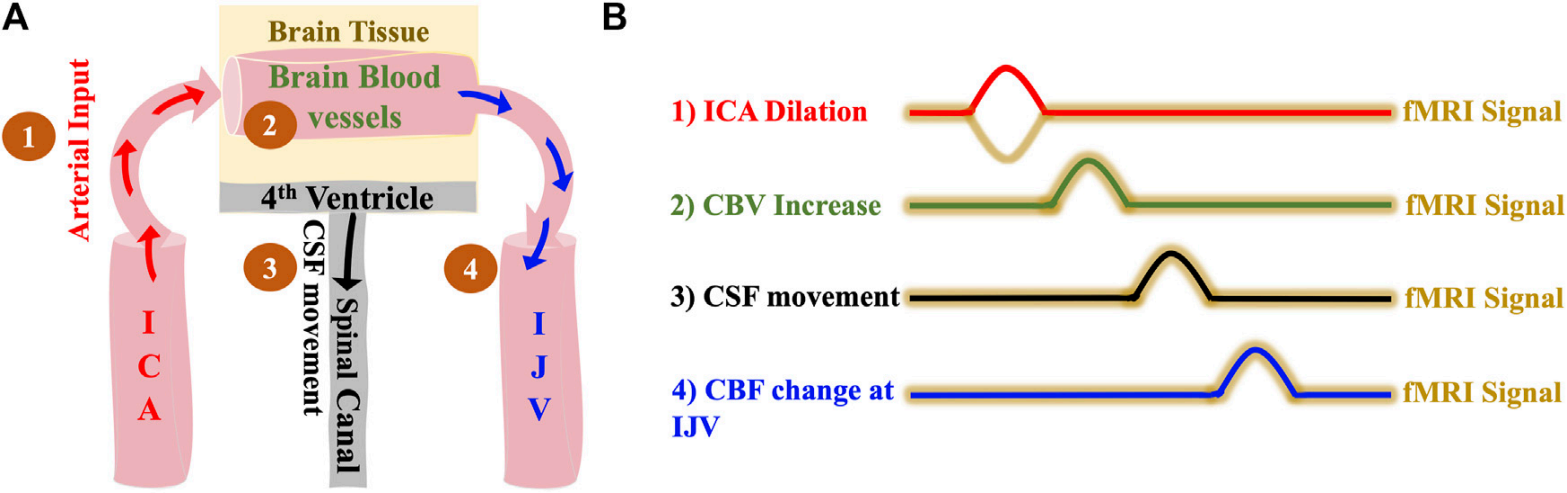
The hemodynamic model of CSF dynamics. **(A)** Model illustrating changes in vessel volume dynamics in the LFO range sequentially starting with 1) Dilation of ICA which leads to 2) Expansion of cerebral blood vessels which finally leads to 3) Caudally directed CSF movement out of the brain and 4) with the same LFOs in the brain travelling to the IJVs with the blood flow **(B)** Illustrative time series representation of changes in vessel volume dynamics that leads to caudally directed CSF movement and corresponding fMRI signals. The crests in the representation indicates positive change/increase. ICA, internal carotid artery; IJV, internal jugular vein; CSF, cerebrospinal fluid; fMRI, functional Magnetic Resonance Imaging.

Based on the model (Figure 1), we expect the following: 1) dilation of the ICAs leads to 2) a CBV increase in the brain which causes 3) a caudally directed CSF movement at the fourth ventricle and leads to 4) a BOLD signal change in IJVs. Since the low frequency blood volume changes progress at the speed of the blood flow (Yao et al., 2019), the delays between ICA signal and CSF movement in the LFO range (0.01–0.1 Hz) should be around 5–6 s in a healthy subject.

#### 2.2.2 Respiration model

Respiration has recently been identified as a major driver of CSF flow, in the context of Monro-Kellie hypothesis. Dreha-Kulaczewski et al. (2017) reported CSF flows upward from the spinal canal in the cranial direction almost instantaneously to compensate for the deep inspiration-induced venous outflow of blood from the brain through the draining veins in the neck (Supplementary Figure S1).

**FIGURE 2:**
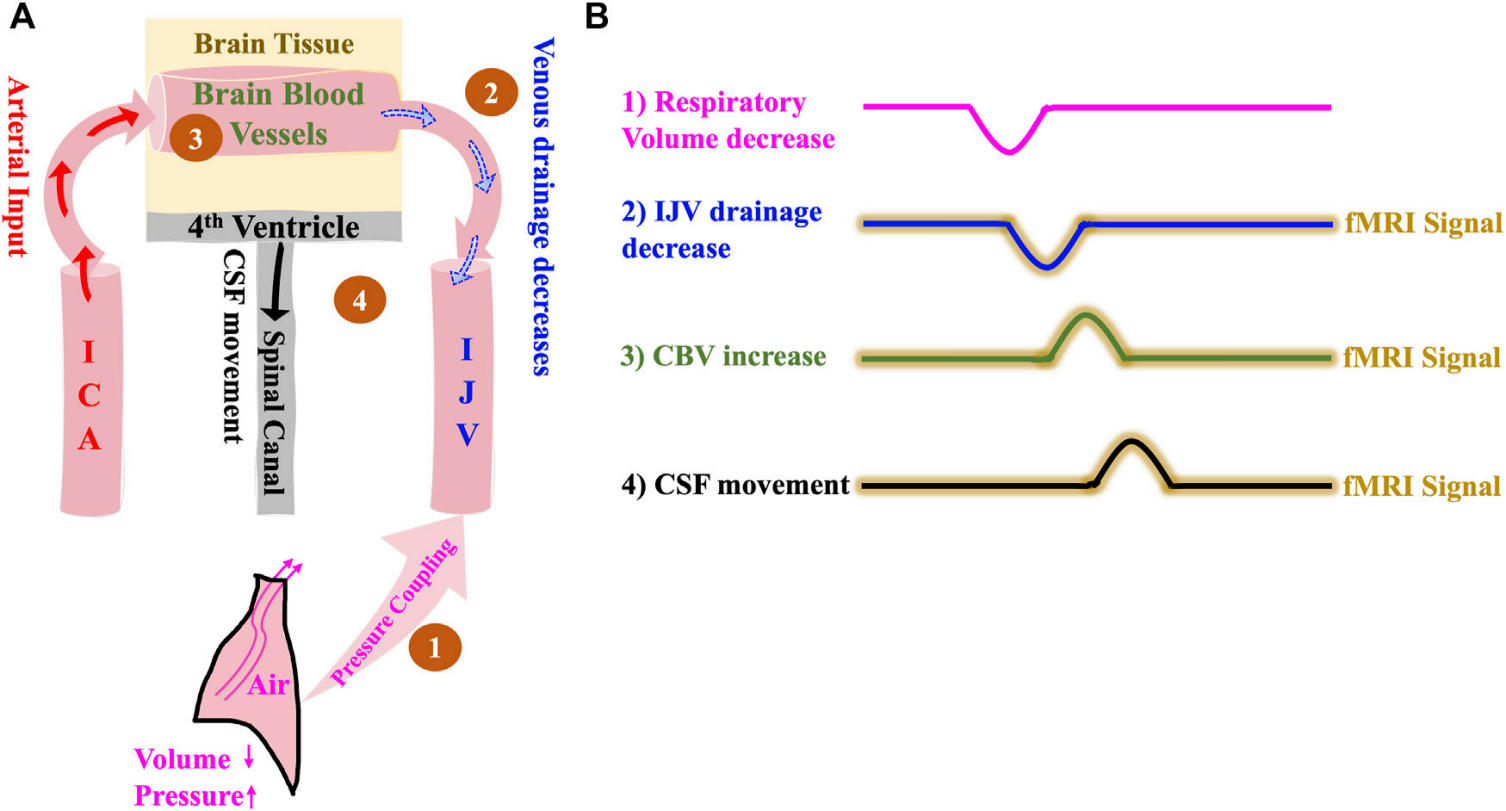
The Respiration model of CSF dynamics. **(A)** Model illustrating how a negative change in intrathoracic volume/positive change in intrathoracic pressure affect CSF dynamics sequentially starting with 1) Pressure coupling to IJV which leads to 2) Reduction in venous drainage in the IJV which in turn leads to 3) Increase in the volume of intracranial blood and finally results in 4) Caudally directed CSF movement out of the brain. **(B)** Illustrative time series representation of intrathoracic pressure-change derived mechanism of CSF flow and corresponding fMRI signals. The crests and troughs in the representation indicates positive change/increase and negative change/decrease respectively. ICA, internal carotid artery; IJV, internal jugular vein; CSF, cerebrospinal fluid; fMRI, functional Magnetic Resonance Imaging.

It is well known that the expiration phase causes the intrathoracic volume to decrease and the intrathoracic pressure to increase, leading to reduced venous return to the heart (Schrauben et al., 2015). In this model, we attempt to validate this mechanism using fMRI data acquired using novel neck scans. During inspiration, decreasing intrathoracic pressure causes increased venous return. As illustrated in Figure 2, the increase in intrathoracic pressure leads to the following: the venous drainage from the cerebral blood vessels into the IJVs decreases, causing cerebral blood vessels to expand (Schrauben et al., 2015) and thereby leading to an increase in the CBV. In addition, the undisturbed arterial input from ICAs might also contribute to this increase in CBV. This then, as per the Monro-Kellie doctrine, immediately leads to a compensatory movement of CSF from the brain into the spinal canal through the fourth ventricle. It is to be noted here that the CSF flow, according to this model, happens only with a change in intrathoracic volume/pressure.

Based on the model (Figure 2), we expect the following: 1) Intrathoracic pressure increase (volume decrease) during expiration causes 2) an IJV drainage decrease which leads to 3) a CBV increase and consequent parenchyma pressure increase that invokes 4) a caudally directed CSF movement at fourth ventricle. We expect that the delays between the detectable signals in the respiratory frequency range (0.2–0.4 Hz) will be no more than 1–2 s, since the venous drainage slows down almost immediately with expiration-related pressure change (Schrauben et al., 2015).

### 2.3. Experimental design

**FIGURE 3:**
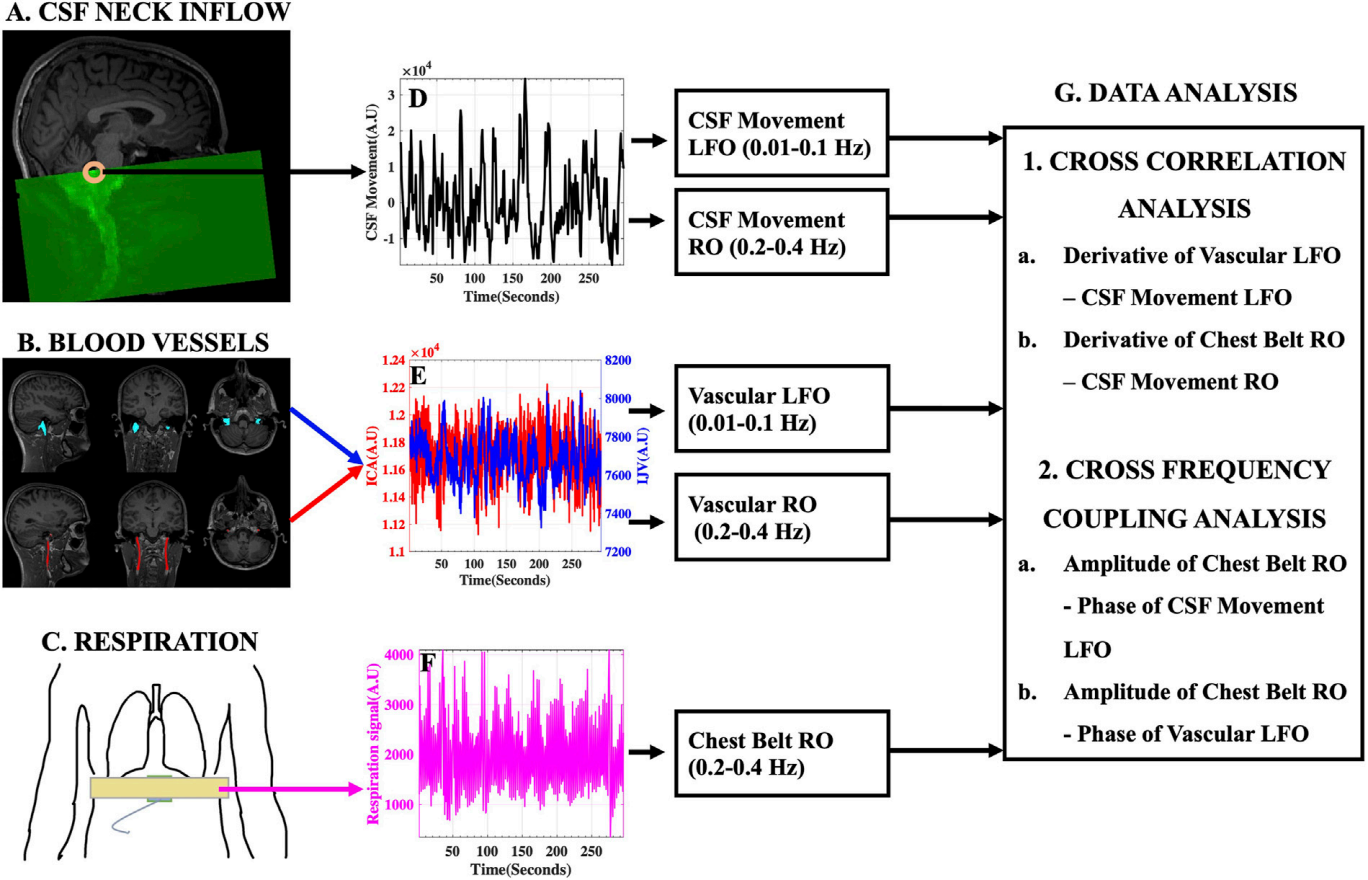
Experimental Design and Data Analysis Stream. **(A)** Typical fMRI scan design illustrating the capture of CSF movement into the neck scan volume utilizing inflow effect and the **(D)** corresponding raw CSF movement signal. **(B)** Large blood vessels of the neck [Left and Right ICA (red) and IJV (blue)] identified and overlaid on structural T1-weighted image and the **(E)** corresponding ICA (red) and IJV (blue) fMRI signals. **(C)** Placement of MRI chest belt and the corresponding **(F)** Raw chest-belt respiratory signal. LFOs and ROs were extracted from CSF and blood vessels and used in the **(G)** data analysis techniques to study the effects on CSF dynamics. AU, Arbitrary Units; ICA, internal carotid artery; IJV, internal jugular vein; CSF, cerebrospinal fluid; LFO, low frequency oscillations (0.01–0.1 Hz); RO, respiratory oscillations (0.2–0.4 Hz).

A pictorial description of the experimental design and data analysis stream is given in Figure 3.

#### 2.3.1 Magnetic resonance imaging data acquisition

All participants were scanned using a 3T SIEMENS MRI scanner (Magnetom Prisma, Siemens Medical Solutions, Erlangen, Germany) with a 64-channel head coil. Structural T1-weighted MPRAGE (Magnetization Prepared Rapid Acquisition Gradient Echo) images were acquired with the following parameters: TR/TE: 2300/2.26 ms, 192 slices per slab, flip angle: 8°, resolution: 1.0 mm × 1.0 mm × 1.0 mm. Structural 3D SPACE (Sampling Perfection with Application optimized Contrasts using different flip angle Evolution) T2-weighted images were acquired with the following parameters: TR/TE: 2800/409 ms, 208 slices per slab, resolution: 0.8 mm × 0.8 mm × 0.7 mm. Resting state functional MRI (fMRI) scans of the neck were acquired using: FOV = 230 mm, acquisition matrix = 92 × 92, 48 slices, voxel size = 2.5 mm × 2.5 mm × 2.5 mm, TR/TE = 960/30.6 ms, echo-spacing = 0.51 ms, flip angle = 35°, multiband acceleration factor = 8, multi-slice mode: interleaved. TR was changed to 440 ms in three participants and to 480 ms in two of them. All participants wore a chest belt (Figure 3C) to measure chest motion.

The fMRI scans of the neck region were designed to capture the caudally directed CSF movement utilizing the inflow effect (Fultz et al., 2019). As illustrated in Figure 3A, by placing the top edge of the scan volume appropriately at the fourth ventricle, an intense CSF flow signal directed into the scan volume can be obtained. Consequently, the slice of interest at the fourth ventricle, was always acquired first in the scan. We demonstrate that this intense single-voxel CSF signal from the edge slice is predominantly of inflow effect origin by comparing it with similar single-voxel signals from the subsequent lower slices in the neck scan volume (Refer to Supplementary Figure S2). We chose to evaluate the CSF movement at the level of the fourth ventricle, since it is the channel through which the CSF exits the brain into the spinal canal. Moreover, the narrow-tapered structure of the fourth ventricle limits the flow of CSF in other directions, thereby strengthening the inflow effect. It is worth noting that the flow-weighted signal in the opposite direction (i.e., CSF flowing towards the brain) is very weak in the top slice of the scan (Figure 3D) and we do not attempt to interpret these small negative signal changes.

#### 2.3.2 Data processing

##### 2.3.2.1 Pre-processing

MR data were processed using FSL [FMRIB Expert Analysis Tool, v6.01; Oxford University, United Kingdom (Jenkinson et al., 2012)] and MATLAB (MATLAB 2020b; The MathWorks Inc., Natick, MA, 2000). Firstly, the CSF signal was extracted from a voxel in the fourth ventricle identified by overlaying the fMRI data over the structural T1-weighted image registered to the fMRI data (Figures 3A,D). Care was taken to identify the signal from the center of the fourth ventricle, in such a way that the CSF signal comes from a voxel with negligible partial-volume effects from surrounding tissues. The fMRI data were only corrected for slice-timing (FSL SLICETIMER) before extracting the single voxel CSF signal. No motion-correction was applied, since it distorts the slice position information required for inflow analysis. Moreover, it would be inaccurate on edge slices where the tissue moves in and out of the imaging volume (Fultz et al., 2019; Yang et al., 2021). However, to confirm that the CSF signals were not corrupted by motion, we assessed and document no significant correlations between motion parameters (FSL MCFLIRT) and the CSF signals (Supplementary Table S1). Having obtained a CSF-only signal from the fourth ventricle without motion correction, motion correction (FSL MCFLIRT) and slice-timing correction (FSL SLICETIMER) were applied to fMRI data used in subsequent analyses.

##### 2.3.2.2 Blood vessel extraction

The left and right ICAs and IJVs were identified using T1-weighted and T2-weighted structural images and these vessel masks (Figure 3B), registered on to the functional space, were used to extract the corresponding fMRI time series (Figure 3E). These methods have been used and validated in previous studies (Tong et al., 2018; Yao et al., 2019; Amemiya, Takao and Abe, 2020).

##### 2.3.2.3 Hemodynamic model verification analysis

The ICA, IJV, and CSF signals were linearly detrended and demeaned. For analysis of the hemodynamic model based on LFOs, the signals were bandpass filtered to the range of 0.01–0.1 Hz, using a zero delay fourth-order Butterworth filter to extract the corresponding LFOs. The LFOs of left and right ICAs and IJVs exhibited high correlations (Right-Left ICAs: 0.57 ± 0.10 and Right-Left IJVs: 0.64 ± 0.13). Hence, the vessel signals of the same kind were averaged for each participant to increase the signal-to-noise ratio (SNR).

As we demonstrated in our previous work (Yang et al., 2021), the changes of CBV rather than CBV itself, are the driving force of CSF movement. Without the CBV changes, no force will be exerted on the ventricles. Therefore, instead of the fMRI signal itself, we used the derivative of the fMRI signal to reflect the preceding intravascular blood volume changes in the ICAs and the corresponding downstream intravascular blood flow changes in the IJVs (Please note that based on previous studies (Tong et al., 2018; Yao et al., 2019), the volume change in the ICAs has been found to be highly correlated with the global CBV change in the brain, and preceding the global CBV by ∼3 s. Similarly, the corresponding downstream flow changes in the IJVs are also highly correlated with the upstream global CBV changes and are delayed by ∼4 s. Since we only scan the neck here, the volume change in the ICAs and corresponding downstream flow changes in the IJVs are used as surrogates of the global CBV. Further, maximum cross-correlation coefficients (MCCCs) and corresponding time delays for each participant were calculated (MATLAB xcorr, maximum lag range: ±15 s) between (1) $\frac{d}{dt}$ (LFO_ICA_) and LFO_CSF_ and (2) $\frac{d}{dt}$ (LFO_IJV_) and LFO_CSF_. The absolute maximum value from the calculated CCCs was identified as the MCCC with its original arithmetic sign. Based on previous research, only the MCCCs above the statistically established threshold of 0.3 (*p*-value <0.01 for positive MCCCs) or below −0.3 (*p*-value <0.01 for negative MCCCs) are considered significant in the LFO range (Hocke et al., 2016; Yao et al., 2019). Accordingly, non-parametric one sample Wilcoxon signed rank test against 0.3/−0.3 was applied on MCCCs in the LFO range, since they were not normally distributed (one sample Kolmogorov-Smirnov test was used to test for normality).

##### 2.3.2.4 Respiration model verification analysis

For analysis of the respiratory model of CSF dynamics, the fMRI signals and the chest belt respiratory signals were bandpass filtered to the range of 0.2–0.4 Hz, using a zero delay fourth order Butterworth filter to extract the corresponding respiratory oscillations (RO). The frequency range of respiration affecting the CSF signals for each participant was identified from the results of cross-spectral analysis between them (Supplementary Figure S3). As in the hemodynamic model verification analysis, the ROs (high correlations between right-left ICAs: 0.37 ± 0.12 and right-left IJVs: 0.48 ± 0.12) from the same kind of vessels were averaged for each participant to increase the SNR.

The difference in pressure between the right atrium of the heart and the brain is the key modulator of venous return from the brain (Schrauben et al., 2015). The intrathoracic pressure changes during respiration are coupled to the right atrial pressure and thereby modulate the venous drainage from the brain (a detailed explanation of how the intrathoracic pressure changes modulate the venous return from the brain is provided in Supplementary Figure S4). To estimate the instantaneous changes in this pressure differential, we use the derivative of chest respiratory oscillations ( $\frac{d}{dt}$ (RO_Chest_)) to represent the instantaneous changes in intrathoracic volume, hence the intrathoracic pressure. MCCCs and corresponding time delays for each participant were calculated (MATLAB xcorr, maximum lag range: ±15 s) between: (1) $\frac{d}{dt}$ (RO_Chest_) and RO_CSF_; (2) $\frac{d}{dt}$ (RO_Chest_) and RO_IJV_; and (3) $\frac{d}{dt}$ (RO_Chest_) and ICA respiratory oscillations (RO_ICA_). In this model, MCCCs and time delays were calculated by forcing the $\frac{d}{dt}$ (RO_Chest_) to lead the other signals, under the assumption that respiration is the driver.

Since the signals in the respiratory range can be highly periodic, there is a risk of spurious cross correlation errors. To reduce false positives on our cross-correlation results in the respiration model, we first limited the cross-correlation window to reflect the fact that $\frac{d}{dt}$ (RO_Chest_) signal always leads the MRI signals (based on the model that respiration initiates the whole process). Second, we sought to establish a statistically significant threshold for the MCCCs to be qualified as physiologically meaningful using existing data. To establish this threshold, we calculated the MCCCs between (1) $\frac{d}{dt}$ (RO_Chest_) and RO_CSF_; (2) $\frac{d}{dt}$ (RO_Chest_) and RO_IJV_; and (3) $\frac{d}{dt}$ (RO_Chest_) and RO_ICA_, after mismatching the signals across participants in random orders. The whole procedure was then repeated 5000 times for each mismatched time-series combination and a distribution of about 55,000 MCCCs were generated in each of the three cases to determine a statistically significant threshold for MCCCs in the respiratory range.

##### 2.3.2.5 Cross–frequency coupling

Cross–frequency coupling (CFC) quantifies the coupling between signals across different frequency bands. In this study, CFC was employed to assess if there is any coupling between RO and LFO bands. Phase-Amplitude Coupling (PAC), a type of CFC, was used to quantify the intensity of CFC between amplitude of RO_Chest_ and phase of LFO_CSF_, LFO_IJV_, and LFO_ICA_, using Mean Vector Length based Modulation Index (Canolty et al., 2006). Statistically significant values of modulation indices between amplitude of RO_Chest_ and phase of LFO_CSF_, LFO_IJV_, and LFO_ICA_ were calculated at 5 percent level of significance as outlined previously (Onslow, Bogacz and Jones, 2011).

## 3 Results

### 3.1 Hemodynamic model

**FIGURE 4:**
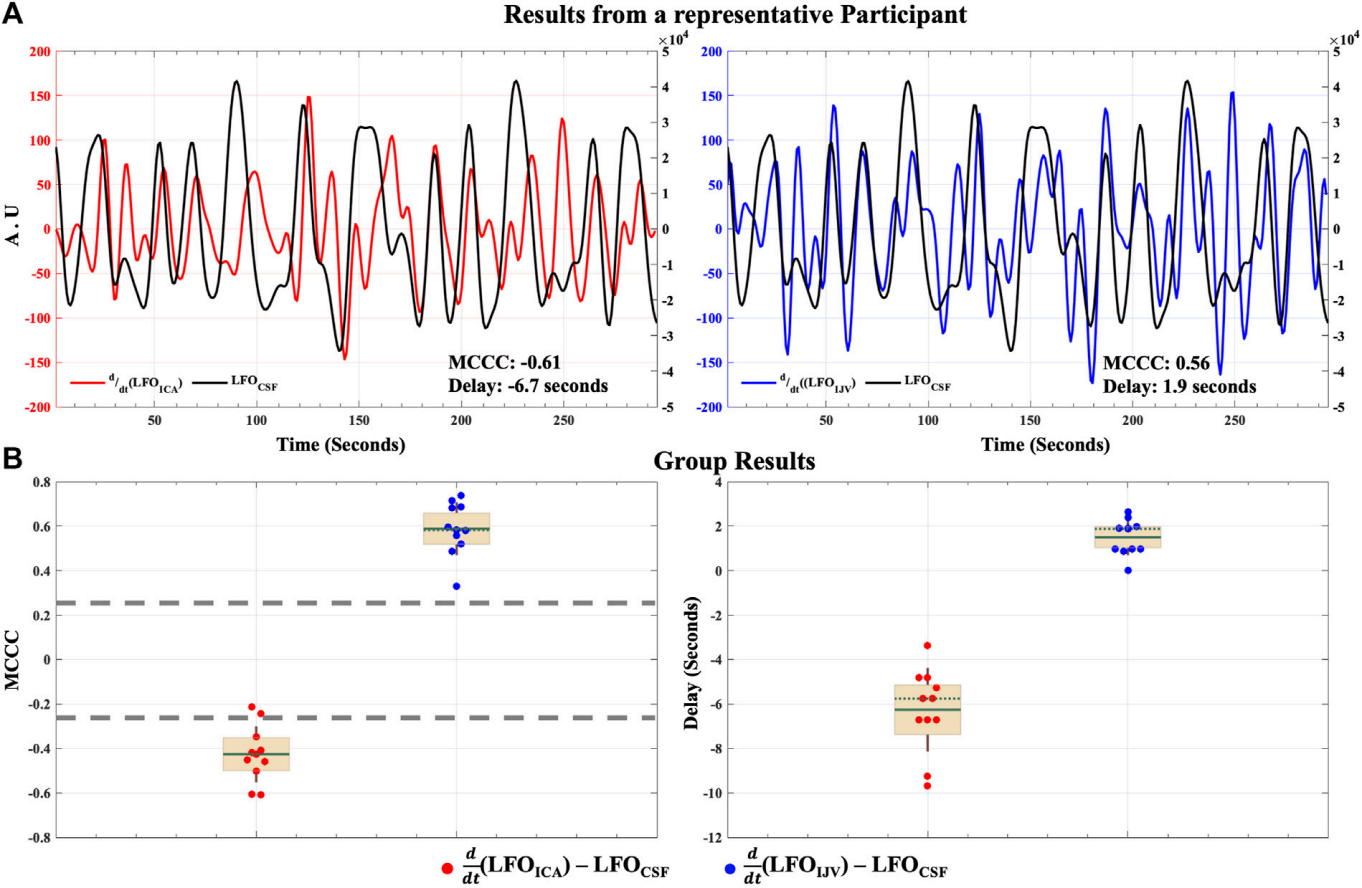
Caudally directed CSF movement in the LFO range. Results of MCCCs and corresponding delays between $\frac{d}{dt}$ (LFO_ICA_) and $\frac{d}{dt}$ (LFO_IJV_) to LFO_CSF_ for **(A)** a representative participant and **(B)** for all participants enrolled in the study. ICA, internal carotid artery; IJV, internal jugular vein; CSF, cerebrospinal fluid; LFO, low frequency oscillation (0.01–0.1 Hz); AU, Arbitrary Units; MCCC, maximum cross-correlation coefficient. In group results **(B)**, the green solid line represents the mean, the green dotted line represents the median, the brown whiskers represent one standard deviation of the raw data points jittered over a 95 percent confidence interval in cream and the gray dashed lines represent the thresholds of statistical significance for MCCCs in the LFO range.

The results of the cross-correlation analysis for the hemodynamic model of CSF dynamics in LFOs are illustrated in Figure 4. MCCCs and corresponding delay between $\frac{d}{dt}$ (LFO_ICA_) and LFO_CSF_ as well as between the $\frac{d}{dt}$ (LFO_IJV_) and LFO_CSF_ from a representative participant can be found in Figure 4A. The data shows that the $\frac{d}{dt}$ (LFO_ICA_) is negatively correlated with LFO_CSF_, with a time delay of -6.7 s. The negative delay indicates that the $\frac{d}{dt}$ (LFO_ICA_) signal leads the LFO_CSF_. An earlier work on resting state fMRI has shown that LFO_ICA_ signal leads the global brain LFO signal by ∼3 s (Tong et al., 2018). It can therefore be inferred that global brain LFO precedes the LFO_CSF_ in the spinal canal by 3-4 s. Across all participants (Figure 4B) there is a significant mean negative correlation of -0.43 ± 0.12 (*p*-value < 0.05) between $\frac{d}{dt}$ (LFO_ICA_) and LFO_CSF_, with a time delay of -6.3 ± 1.8 s.

The MCCCs and corresponding delay times between $\frac{d}{dt}$ (LFO_IJV_) and LFO_CSF_, for the same representative participant (Figure 4A) yields a positive correlation with a delay of 1.92 s. The positive time delay indicates that the $\frac{d}{dt}$ (LFO_IJV_) signal lags the LFO_CSF_. Across all participants (Figure 4B) there is a significant mean positive correlation of 0.59 ± 0.11 (*p*-value < 0.05) between $\frac{d}{dt}$ (LFO_IJV_) and LFO_CSF_, with a time delay of 1.5 ± 0.79 s.

### 3.2 Respiration model

#### 3.2.1 Maximum cross-correlation coefficients distribution in the respiratory oscillations range of frequencies

**FIGURE 5:**
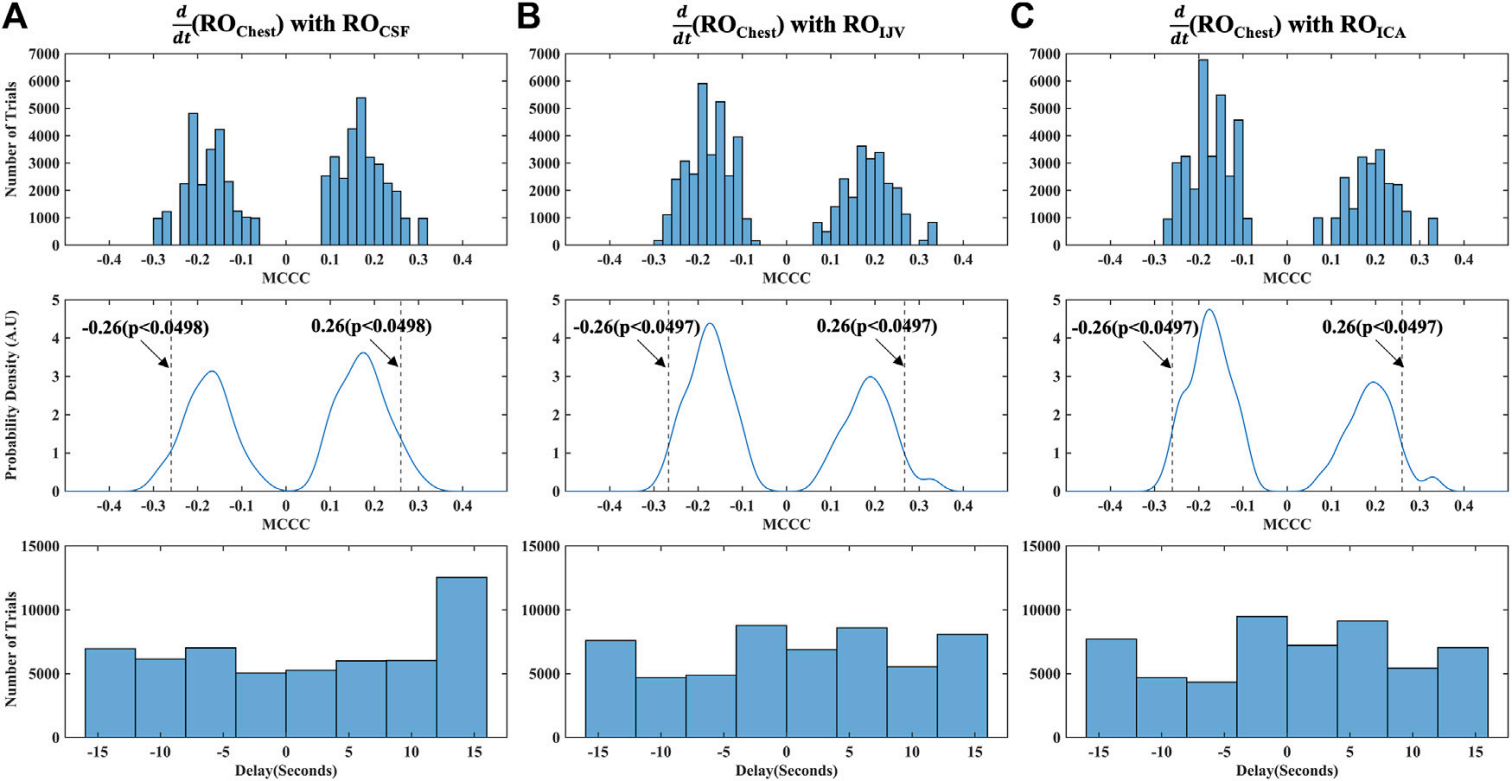
Distribution of MCCCs and corresponding delays calculated from randomly mismatched **(A)** $\frac{d}{dt}$ (RO_Chest_) and RO_CSF_; **(B)** $\frac{d}{dt}$ (RO_Chest_) and RO_IJV_ and **(C)** $\frac{d}{dt}$ (RO_Chest_) and RO_ICA_. ICA, internal carotid artery; IJV, internal jugular vein; CSF, cerebrospinal fluid; RO, respiratory oscillation (0.2–0.4 Hz); AU, Arbitrary Units; MCCC, maximum cross-correlation coefficient.

The distribution of all the spurious MCCCs (and corresponding delays) calculated from mismatched RO range time series, i.e., between mismatched $\frac{d}{dt}$ (RO_Chest_) and RO_CSF_; $\frac{d}{dt}$ (RO_Chest_) and RO_IJV_ and $\frac{d}{dt}$ (RO_Chest_) and RO_ICA_ are displayed respectively in Figures 5A–C. The MCCCs are mostly clustered around −0.18 and 0.18 (Figure 5, top row). In addition, the *p*-values from these distributions show that MCCCs less than -0.26 (for negative MCCCs) or greater than 0.26 (for positive MCCCs) are statistically significant (*p*-value<0.05) (Figure 5, middle row). Hence, the MCCC’s in the RO range were tested against 0.26/-0.26 using the Wilcoxon signed rank test (Data were not normally distributed (one sample Kolmogorov-Smirnov test was used to test for normality). The delays (Figure 5, bottom row) are evenly distributed across the cross-correlation window, which is consistent with the randomization in data pairing. A comparatively higher concentration at the boundary (12–15 s in Figure 5A, bottom row), arises from delays which are outside the cross-correlation search window.

As a final step to ensure that our cross-correlation results in the RO range were not spurious errors arising from the periodicity of the signals, we also calculated the MCCCs and delays between mismatched halves of RO range time series data for each participant, i.e., between the first half of $\frac{d}{dt}$ (RO_Chest_) and second half of each of the RO_CSF_, RO_IJV_, and RO_ICA_ time series in turn (Supplementary Figure S5). The results fall mostly outside the significant range of MCCCs, thus validating our findings in the respiration model.

#### 3.2.2 Caudally directed Cerebrospinal Fluid movement in the respiratory oscillations range

**FIGURE 6:**
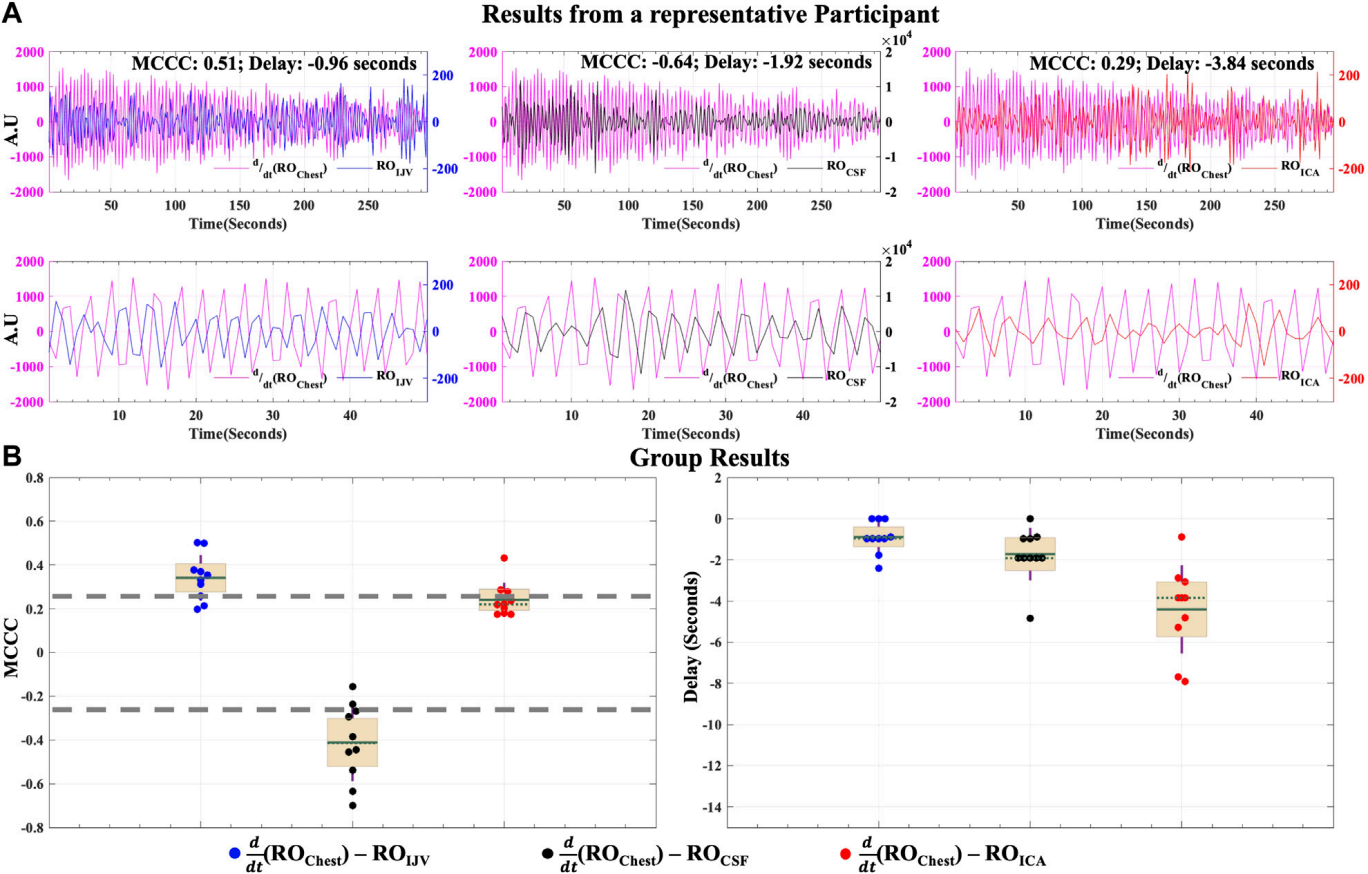
Caudally directed CSF movement in the RO range. Results of MCCCs and corresponding delays between $\frac{d}{dt}$ (RO_Chest_) to RO_CSF_, RO_IJV_ and RO_ICA_ for **(A)** a representative participant (entire signal in top row and first 50 s in bottom row to illustrate the correlations) and **(B)** for all participants enrolled in the study. ICA, internal carotid artery; IJV, internal jugular vein; CSF, cerebrospinal fluid; RO, respiratory oscillation (0.2–0.4 Hz); AU, Arbitrary Units; CCC, cross-correlation coefficients; MCCC, maximum cross-correlation Coefficient. In group results **(B)** the green solid line represents the mean, the green dotted line represents the median, the brown whiskers represent one standard deviation of the raw data points jittered over a 95 percent confidence interval in cream and the gray dashed lines represent the thresholds of statistical significance for MCCCs in the RO range.

Figure 6A shows the MCCCs and the corresponding time delays between $\frac{d}{dt}$ (RO_Chest_) and RO_CSF_, $\frac{d}{dt}$ (RO_Chest_) and RO_IJV_ and $\frac{d}{dt}$ (RO_Chest_) and RO_ICA_ from a representative participant. As postulated in the respiratory model, the $\frac{d}{dt}$ (RO_Chest_) is negatively correlated to RO_CSF_, with a time delay of −1.92 s. Here, the negative value of time delay indicates that the $\frac{d}{dt}$ (RO_Chest_) leads the RO_CSF_. For the same participant (illustrated in Figure 6A), $\frac{d}{dt}$ (RO_Chest_) is positively correlated with the RO_IJV_, leading it by 0.96 s. From these two results, we can infer that the RO_IJV_ leads the RO_CSF_ by around a second. For the group (Figure 6B), $\frac{d}{dt}$ (RO_Chest_) is significantly negatively correlated to RO_CSF_ (MCCC: -0.41 ± 0.17, *p*-value < 0.05), leading it by 1.73 ± 1.28 s, and is also significantly positively correlated to RO_IJV_ (MCCC: 0.35 ± 0.11, *p*-value < 0.05), leading it by 0.89 ± 0.78 s (Mean and standard deviation values here were calculated after excluding the results from one participant that exhibited unrealistic delays greater than 10 s, that does not fit into the proposed model, due to lower SNR of that participant’s chest-belt signal). For the group, we can again infer that RO_IJV_ leads RO_CSF_ by around a second. Taken together with the significant difference between these average delay values (*p*-value < 0.05), these results confirm that RO_Chest_ changes affect IJVs first, leading to a change in the CBV through the mechanism of reduced venous drainage, thereby leading to the caudally directed CSF movement into the spinal canal.

To determine if respiration might also affect the arterial supply within a time frame short enough to induce an effect measurable under the proposed venous return model, we also calculated the correlations between $\frac{d}{dt}$ (RO_Chest_) and RO_ICA_. However, the results were not consistent, with low correlation mean values of 0.24 ± 0.07 (*p*-value > 0.05) and delays of around −4 s (*p*-value < 0.05) that are outside of the time-frame of communication predicted by the respiration model.

### 3.3 Coupling between respiration and low-frequency oscillations

**FIGURE 7:**
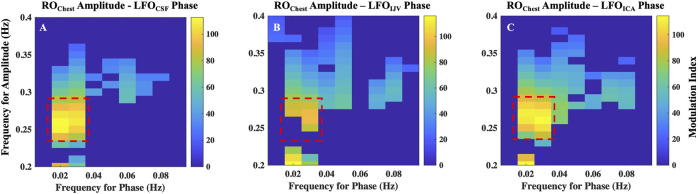
Cross-frequency coupling between respiration and LFOs. Results of coupling quantified by modulation index, between amplitude of RO_Chest_ signal and the phase of **(A)** LFO_CSF_, **(B)** LFO_IJV_ and **(C)** LFO_ICA_ signals for a representative. Highlighted regions represent frequencies with maximal coupling. ICA, internal carotid artery; IJV, internal jugular vein; CSF, cerebrospinal fluid; LFO, low frequency oscillations (0.01–0.1 Hz); RO, respiratory oscillations (0.2–0.4 Hz)

The results of cross-frequency coupling between amplitude of RO_Chest_ signal and the phase of LFO_CSF_, LFO_IJV_ and LFO_ICA_ signals for a representative participant are delineated in Figures 7A–C, respectively. The plots only show the values of CFC at 5% level of significance (Onslow, Bogacz and Jones, 2011).

The highest coupling is between amplitude of RO_Chest_ signal in the frequency range 0.25–0.3 Hz with the phases of LFO signals in the frequency of range 0.016–0.035 Hz. It is to be noted that the range 0.25–0.3 Hz is the peak frequency range of respiration for the given participant. All participants exhibited a similar pattern, having the maximum coupling with LFO signals at the peak frequency range of the respiration for the individual. However, the LFO range of maximal coupling varies across subjects (Supplementary Figure S6). This implies that respiration mostly affects a narrow range of frequencies in the LFO range.

## 4 Discussion

In this study, we have demonstrated several significant findings. First, we show that the dynamics of brain fluids can be assessed from the neck, by studying the interrelationships between major neck blood vessels and the CSF movement at the fourth ventricle. Second, we validate via fMRI neck scan that both low frequency hemodynamics (0.01–0.1 Hz) and respiration (0.2–0.4 Hz) influence CSF movement in the respective frequency ranges, albeit through different mechanisms. Finally, we show that there exists a cross-frequency coupling between the amplitude of respiration and phase of LFOs found in CSF, IJV, and ICA. Below, we provide a physiological interpretation of these findings and their implications for CSF dynamics. In addition, we discuss the potential clinical impact of our results.

### 4.1 Effects of low frequency hemodynamics

In this study, we investigated the vascular LFOs (0.01–0.1 Hz), which originated outside the brain, as a possible driving force responsible for CSF movement in humans. Our results demonstrate that the derivative of these low frequency hemodynamic oscillations extracted from ICA and IJV in the neck, correlate with LFO_CSF_ directed toward the spinal canal within a physiologically relevant time frame. This is the first study to establish the fMRI temporal relationship between ICA and caudally directed CSF movement in the LFO range.

Evidence from our prior resting state fMRI study in awake humans illustrate that LFOs within the brain (i.e., global mean fMRI signal) represent the global cerebral blood volume fluctuations, whose collective force on the lateral ventricles results in CSF movement (Yang et al., 2021). Results from the current hemodynamic model support and extend this phenomenon by tracing these LFOs at the ICA. We show that these LFOs enter the brain through the ICAs and take an average of 6 s to have the accumulative CBV effects (in the brain), finally culminating in caudally directed CSF movement observed at the fourth ventricle (Figure 4). Lastly, these LFOs appear at the IJV seconds later.

Various physiological mechanisms have been suggested as the possible origin of vascular LFOs. Variations in blood CO_2_ (a potent vasodilator) is a possible major global source of vascular LFOs, as it is also known to dilate vessels as it travels along with blood (Wise et al., 2004; Golestani et al., 2015). Vasomotion is another potential source of LFOs (He et al., 2018; Noordmans et al., 2018). Results from two recent studies using mathematical modeling (Aldea et al., 2019) and *in vivo* two photon microscopy in rodents (van Veluw et al., 2020) support the idea that local vasomotion generated from vascular smooth muscle cells might be driving CSF dynamics. Other potential candidates such as mayer waves (Julien, 2006), volume changes in veins caused by dilation of upstream arteries (windkessel effect) (Buxton, Wong and Frank, 1998), autoregulatory mechanisms (Müller and Österreich, 2014) may also be contributing to these low-frequency blood volume changes. Although our study establishes the influence of low frequency hemodynamics in driving CSF, the exact origin of these LFOs is not clear.

Lastly, it is worth noting that there are various fMRI endogenous contrasts used in this study, which include the inflow effect at the fourth ventricle, the BOLD effect at the draining veins, and an obscure BOLD effect at ICAs. The ICAs in healthy subjects carry blood with ∼100% oxygenation. However, a weak positive BOLD effect might arise through variations in arterial saturation (Aso et al., 2019). Alternatively, a weak negative BOLD effect might arise via changes in arterial diameter since fully saturated arterial blood has a lower magnetic susceptibility than tissues. Given the negative correlations between the derivative of LFO_ICA_ (i.e., representing dilation) and LFO_CSF_, the most likely contrast in ICA is a partial volume effect arising from changes in arterial diameter, producing an fMRI signal inversely correlated with arterial blood volume (Tong et al., 2018; Yao et al., 2019; Amemiya, Takao and Abe, 2020).

### 4.2 Effects of respiration

Respiration is another important mechanism that has been suggested as a potential engine for CSF movement (Klose et al., 2000; Friese et al., 2004; Yamada et al., 2013; Chen et al., 2015; Dreha-Kulaczewski et al., 2017). Our results point to the possibility of a highly interdependent and tightly communicating CSF-venous system (i.e., in the respiratory frequency range). In detail, our results document a negative change in intrathoracic volume (i.e., a positive change in intrathoracic pressure) leads to a reduction in venous drainage from the brain observed within a second at the jugulars, further leading to an increase in blood volume in the brain (due to reduced drainage) and finally resulting in a compensatory outward movement of CSF from the brain within an average time of 2 s from the start of the cycle. The observed time delays, although small, show that respiration indeed influences CSF dynamics through the venous system in a sequential and continual fashion.

We further explored the possibility that the intrathoracic volume/pressure changes also affect the arterial system. The results showed that these signals correlate weakly at time delays much longer (∼4.4 s) than that taken by respiration to affect the venous system (<1 s). This is likely to be due to different mechanisms by which respiration couples with venous and arterial systems and to the relatively lower SNR of fMRI signal in the arteries. Our respiratory model (Figure 2) did not include a specific route by which changes of intrathoracic volume/pressure might couple with ICA but there are a few candidate mechanisms, including variations in minute ventilation (the volume of gas inhaled/exhaled per minute) leading to changes in arterial oxygen saturation and arterial CO_2_ concentration. The weak positive correlation of chest motion and RO_ICA_, with chest motion leading by around 4.4 s, would fit a standard BOLD signal model in which increased minute ventilation (larger breaths) leads to greater arterial oxygen saturation and dilution of the small concentration of arterial deoxyhemoglobin. Variation of arterial deoxyhemoglobin has been proposed by Aso et al. (2019) as one possible source of LFOs. Future work might expand the respiratory model to include minute ventilation as an independent measure. Together with measurements of expired CO_2_ it might then be possible to determine a precise mechanism for the weak coupling between chest motion and RO_ICA_.

### 4.3 Coupling and relative effects

The results from our study have shown that both low frequency hemodynamics and respiration generate fluctuations of CSF flow in the fourth ventricle in humans. We found that the amplitude of respiration measured from the chest is tightly coupled with the phases of LFOs found in CSF movement. We also found a similar coupling between respiration amplitude and the phases of vascular LFOs extracted from ICAs and IJVs. These results point to the possibility that the depth of respiration (i.e., minute ventilation) contributes to the changes in blood vessel volume dynamics that occurs in LFO range. This concept is physically plausible, since depth of respiration is capable of inducing changes in blood vessel volume dynamics through intrathoracic pressure changes as well as CO_2_ concentration changes (Chang and Glover, 2009). As suggested by the results from cross-frequency coupling, it is possible that respiration exerts its effects on CSF dynamics not only through the pressure-related venous return changes in the RO range, but also through the blood gas related vessel volume changes, within a narrow range of low frequency oscillations (0.0175–0.028 Hz) in CSF signals (Supplementary Figure S7). On average, respiration explained 73.46% ± 15.83% of variability in the LFO range of 0.0175–0.028 Hz. Natural variations in blood CO_2_ during respiration could be thought of as primarily contibuting to this narrow range of LFOs, consistent with a previous report that the end-tidal CO_2_ fluctuations during rate-controlled resting state breathing were significantly correlated to BOLD fMRI signal fluctuations at ∼0.03 Hz (Birn et al., 2006).

Finally, it is imperative to figure out the relative contributions of each of these mechanisms in regulating CSF dynamics. A power spectrum analysis of the caudally directed CSF signals observed at the fourth ventricle showed that LFOs represent the major component of the signal in all of the participants in this study. In fact, the percent of power in the LFO range is significantly higher than the percent of power in the normal respiration range (Supplementary Figure S8). On average, about 67 percent of power in CSF signal comes from the low frequency oscillations, whereas only about 15 percent of power is present in the respiration range. However, this does not entirely mean that respiration has a negligible effect on CSF movement. CSF flow has also been suggested to be pulsatile in nature, driven by cardiac cycle (Mestre et al., 2018). Central venous pressure changes related to cardiac cycle (Zamboni et al., 2020; Beggs et al., 2021), might also be a contributing factor. However, this mechanism has been identifed through mathematical modelling to be too weak to become the principal force driving CSF (Asgari, De Zélicourt and Kurtcuoglu, 2016; Diem et al., 2017). In our study, cardiac pulsations (>0.6 Hz) were sampled in only 5 participants explained only an average of 11 percent of the power in the caudally directed CSF signals observed at the fourth ventricle (See Supplementary Note S2 and Supplementary Figure S9).

### 4.4 Clinical impact

This study demonstrated that close coupling exists between fluid dynamics in the brain and that this valuable information can be extracted from a neck scan. This opens the possibility of developing a simple neck scan sequence or applying other imaging modalities (e.g., Doppler ultrasound or Near Infrared Spectroscopy) to get indirect, or inferred, CSF measures from the blood vessels in the neck. Such a simple fMRI sequence would also be superior to other conventional methods like Phase contrast MRI (PCMRI) for the following reasons: First, signal acquired from PCMRI is gated to the cardiac cycle—i.e., CSF flow values quantified were averaged over multiple cardiac cycles. For this reason, effects of the physiological frequencies (i.e., LFOs and ROs) other than the cardiac frequency on CSF movement cannot be assessed. Second, simultaneous assessment of signals from all three locations studied here (i.e., ICAs, IJVs, and CSF) cannot be performed accurately, since the appropriate velocity encoding “VENC” parameter for the fluid flow assessment in these three locations are different. Any deviations in this parameter from the optimal value for each location leads to low signal or aliasing artifacts, rendering the recording unreliable (Korbecki et al., 2019). We caution, however, that the first step towards such a clinical tool would be to repeat the current study in patient groups, in case abnormal vascular or CSF dynamics, as found in stroke and hydrocephalus patients, respectively, reduces or eliminates the relationships we observe in healthy subjects. In addition, the study also validated the mechanism of respiration in the CSF movement and its relationship with that of LFOs. These findings can inform the development of clinically relevant breathing tasks that might further couple with LFOs and enhance CSF flow in patients where low natural flow may be a concern, e.g., due to insomnia.

### 4.5 Limitations and future studies

A limitation of this study is the lack of a simultaneous end-tidal CO_2_ measurement. This would have helped to separate the contributions of respiration to CSF dynamics from both pressure changes and CO_2_ changes. The method used in our study was also unable to simultaneously detect the CSF movement into the brain. Another minor limitation is the lower fMRI temporal resolution that made it impossible to sample the cardiac pulsations in 6 participants. Finally, it would have been interesting to study the CSF movement under lowered/elevated vascular (heart rate, blood pressure) and respiratory (resting state vs. hyper/hypoventilation) rate conditions. Future studies will incorporate simultaneous measurement of end-tidal CO_2_ values and effects of the aforementioned conditions on CSF movement.

## 5 Conclusion

In conclusion, the results of our study illustrate that valuable brain fluid dynamic information can be obtained from a neck scan, from which it was found that multiple physiological forces contribute to driving CSF movement in humans. Two simple yet distinct mechanisms simultaneously regulating caudally directed CSF movement in humans, based on 1) low frequency hemodynamics from the neck blood vessels (ICA and IJV) and 2) respiration were validated. Cross-frequency coupling between these motive forces were also identified. The capability of the CSF system to respond to multiple physiological forces at the same time may help unveil the pathological mechanisms behind CSF flow related disorders.

## Data Availability

The raw data supporting the conclusion of this article will be made available by the authors, without undue reservation.
