# Supplementary material for: Human CSF movement influenced by vascular low frequency oscillations and respiration
Source: Front Physiol. 2022 Aug 19;13:940140. doi: 10.3389/fphys.2022.940140 (PMC9437252; doi:10.3389/fphys.2022.940140)
Supplement: Supplementary file 1 [file DataSheet1.docx]

Supplementary Material

Table S1: Correlations between motion parameters (FSL MCFLIRT) and caudally directed CSF signals for each participant. From the results, it can that there exist no noteworthy correlations between motion parameters and the CSF signals. This therefore confirms that the CSF signals extracted from the fMRI scans were not affected by motion. r(x-z): MCFLIRT estimated rotations; t(x-z): MCFLIRT estimated translations.


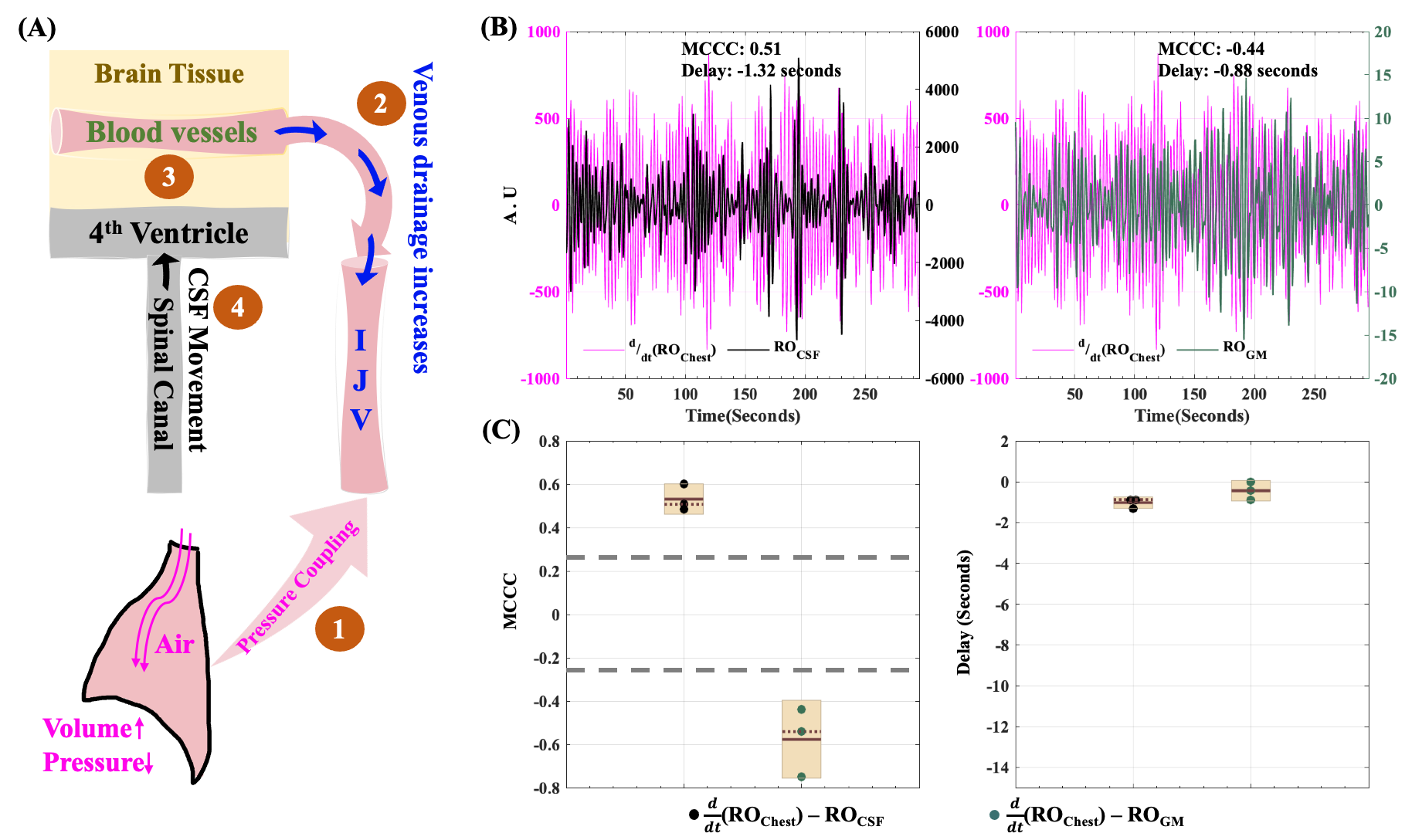


Figure S1: CSF movement to the brain and Respiration. Cranially directed CSF movement to the brain was captured in 3 participants by performing a brain scan with the fourth ventricle still maintained at the first slice of the scan volume. (A) Model illustrating how a positive change in intrathoracic volume/negative change in intrathoracic pressure affect CSF dynamics sequentially starting with 1) Pressure coupling to IJV which leads to 2) Increase in venous drainage in the IJV which in turn leads to 3) Decrease in the volume of intracranial blood and finally results in 4) Cranially directed CSF movement into the brain. Based on this relationship, the d/dt(RO_Chest_) should be positively correlated with cranially directed RO_CSF_ and negatively correlated with global brain-RO (RO_GM_). Results of MCCCs and corresponding delays between d/dt(RO_Chest_) to RO_CSF_, and RO_GM_ for (B) a representative participant and (C) for all three participants with the brain scans. It can be seen that as predicted by the model, the d/dt(RO_Chest_) leads the RO_CSF_ by an average of 1.03±0.25 seconds with an average MCCC of 0.53±0.06 and leads the RO_GM_ by an average time delay of 0.44±0.44 seconds and an average MCCC of -0.57±0.16. The negative delays indicate that the d/dt(RO_Chest)_ signal leads the others. Cross-correlation calculations were performed by forcing the d/dt(RO_Chest_)signal to lead, under the assumption that respiration is the driver to the observed GM and CSF responses in the respiratory frequency range. IJV – Internal Jugular Vein; CSF - Cerebrospinal Fluid; RO – Respiratory Oscillations (0.2 Hz – 0.4 Hz); A.U – Arbitrary Units; CCC – Cross-Correlation Coefficients; MCCC – Maximum Cross-Correlation Coefficient. In group results (B), the brown solid line represents the mean, the brown dotted line represents the median, the brown whiskers represent one standard deviation of the raw data points jittered over a 95 percent confidence interval in cream.

Figure S2: Demonstration of CSF movement signal captured by inflow effect. It can be seen that an intense caudally directed single-voxel CSF movement signal is captured from the edge slice (i.e., slice 1 in the figure), arising from inflow effect. At the same time, the intensity of similar single-voxel signals from subsequent lower slices (i.e., slices 2 and 3) is much lower due to lower sensitivity of these slices to inflow effect. This is because the inflow effect would be reduced since the CSF fluid particles has to move further down into the scan volume to reach these slices and (has already encountered the RF pulses) therefore induce a signal of lower intensity. These results confirm that the caudally directed single-voxel CSF movement signals captured from the edge slices are predominantly originating from inflow effect. A.U – Arbitrary Units; CSF - Cerebrospinal Fluid.

**
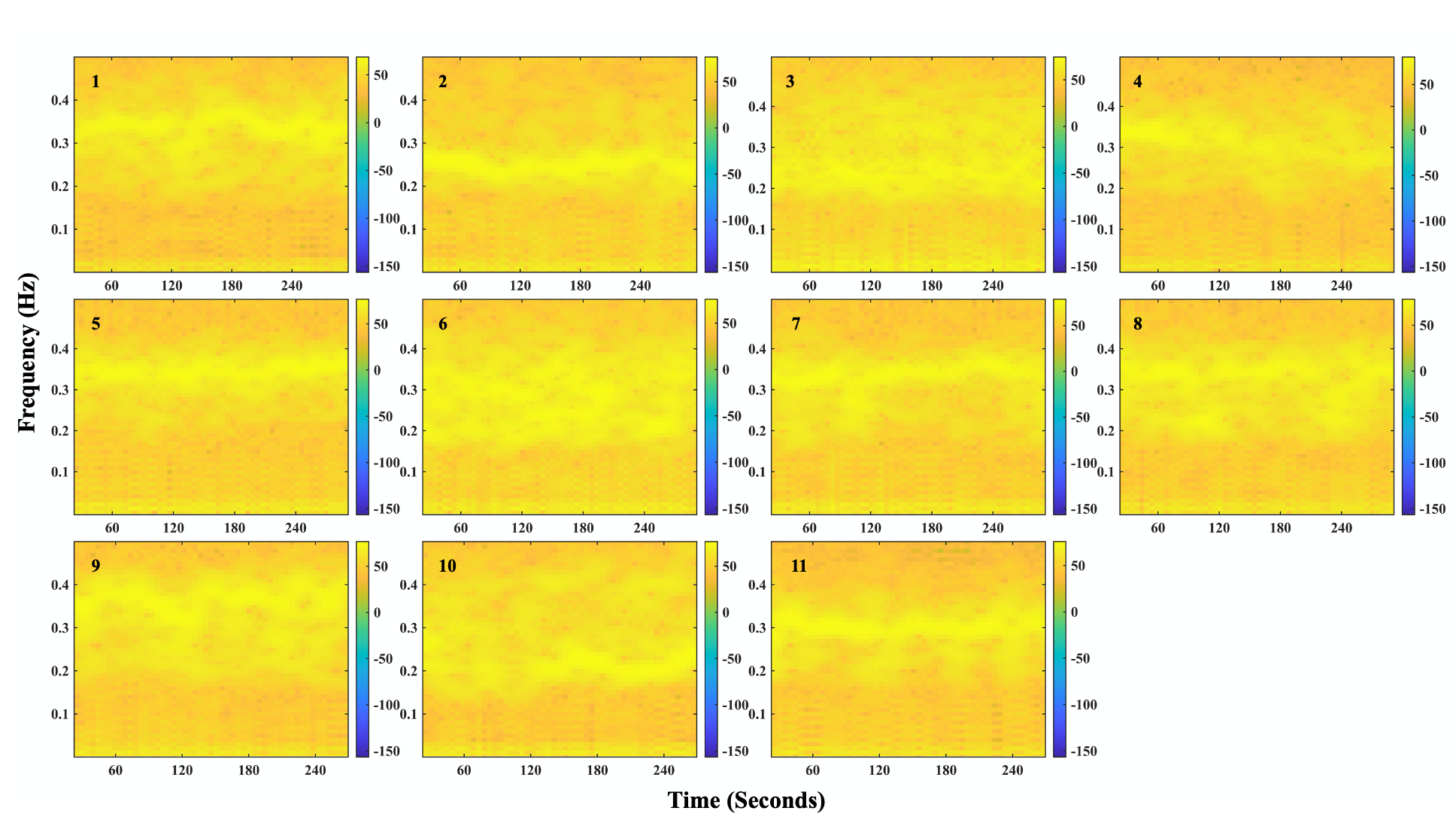
** Figure S3: Cross-Spectral analysis of caudally directed CSF signals and Chest-belt respiration signals. The figure illustrates the results of cross-spectrogram between raw CSF signals and chest-belt respiratory oscillations for all the 11 participants enrolled in this study. It can be seen that the maximum coherence between the signals occur at the normal respiratory range of 0.2 Hz – 0.4 Hz throughout the entire scan duration of 5 minutes, in all participants. The cross-spectrograms were calculated using the MATLAB function ‘xspectrogram’ with window lengths of 50 seconds and a 90 percent overlap. Plots are numbered according to participant number. Colorbars represent power/frequency (dB/Hz).


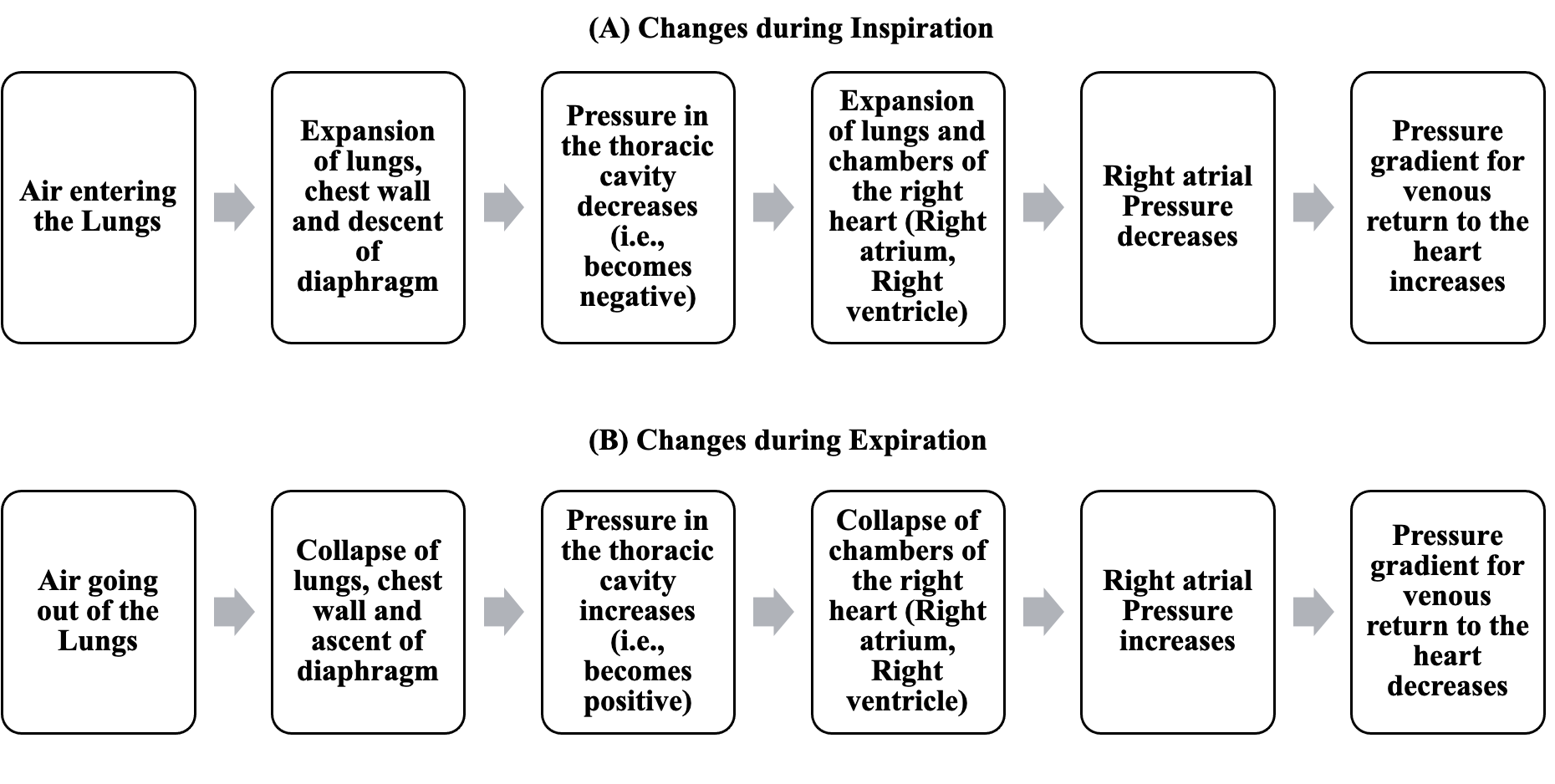


Figure S4: Coupling of intrathoracic pressure changes during respiration to venous return. (A) During inspiration, entry of air into the lungs, leads to simultaneous expansion of lungs, chest wall and descent of the diaphragm. As a result, intrathoracic pressure (i.e., pressure in the thoracic cavity between the organs – lungs, heart and chest wall) decreases (i.e., becomes negative). This leads to further expansion of the lungs and the chambers of the right heart (right atrium and right ventricle). This in turn leads to a decrease in the right atrial pressure and thereby in an increase of the pressure gradient between right atrium and the brain blood vessels, aiding the venous return from the brain to the right atrium. On the other hand (B) during expiration, the reverse occurs. That is, passage of air out of the lungs, leads to simultaneous collapse of lungs, chest wall and ascent of the diaphragm. As a result, intrathoracic pressure increases (i.e., becomes positive). This leads to further collapse of the lungs and the chambers of the right heart (right atrium and right ventricle). This in turn leads to an increase in the right atrial pressure and thereby in a decrease of the pressure gradient between right atrium and the brain blood vessels, reducing the venous drainage from the brain to the right atrium.


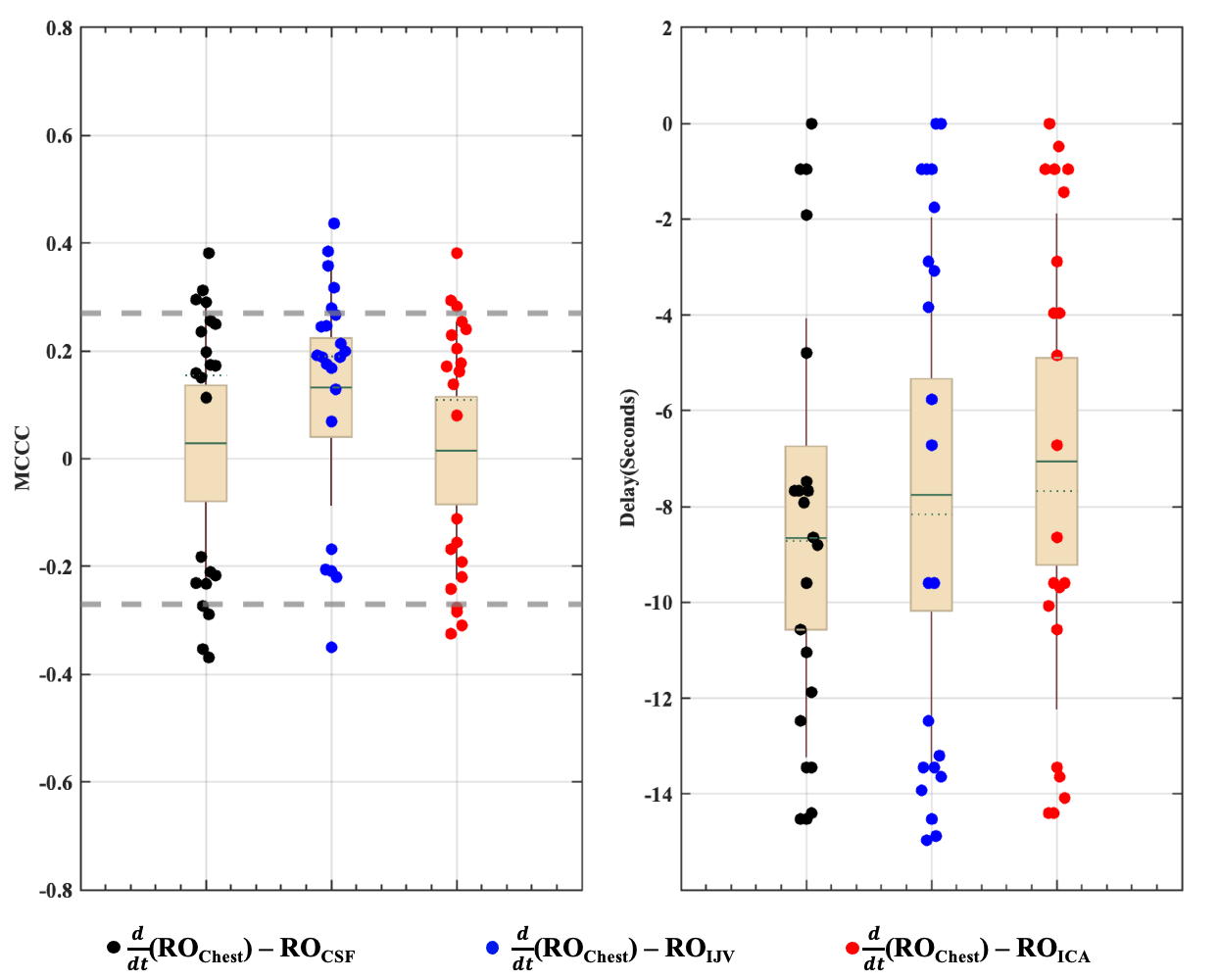


Figure S5: MCCCs and delays between mismatched halves of RO range time series data for each participant. In order to ensure that our cross-correlation results in the RO range were not spurious errors arising from the periodicity of the signals, we also calculated the MCCCs and delays between mismatched halves of RO range time series data. For example, the MCCC and the corresponding time delay was calculated between the first half of d/dt(RO_Chest_) and second half of RO_CSF_/RO_IJV_/RO_ICA_ for each participant. The results illustrate that the spurious MCCCs cluster outside the range of statistical significance(-0.26<MCCC<0.26) in the RO range, thereby validating that our results are not due to spurious errors. ICA – Internal Carotid Artery; IJV – Internal Jugular Vein; CSF - Cerebrospinal Fluid; RO – Respiratory Oscillations (0.2 Hz – 0.4 Hz).


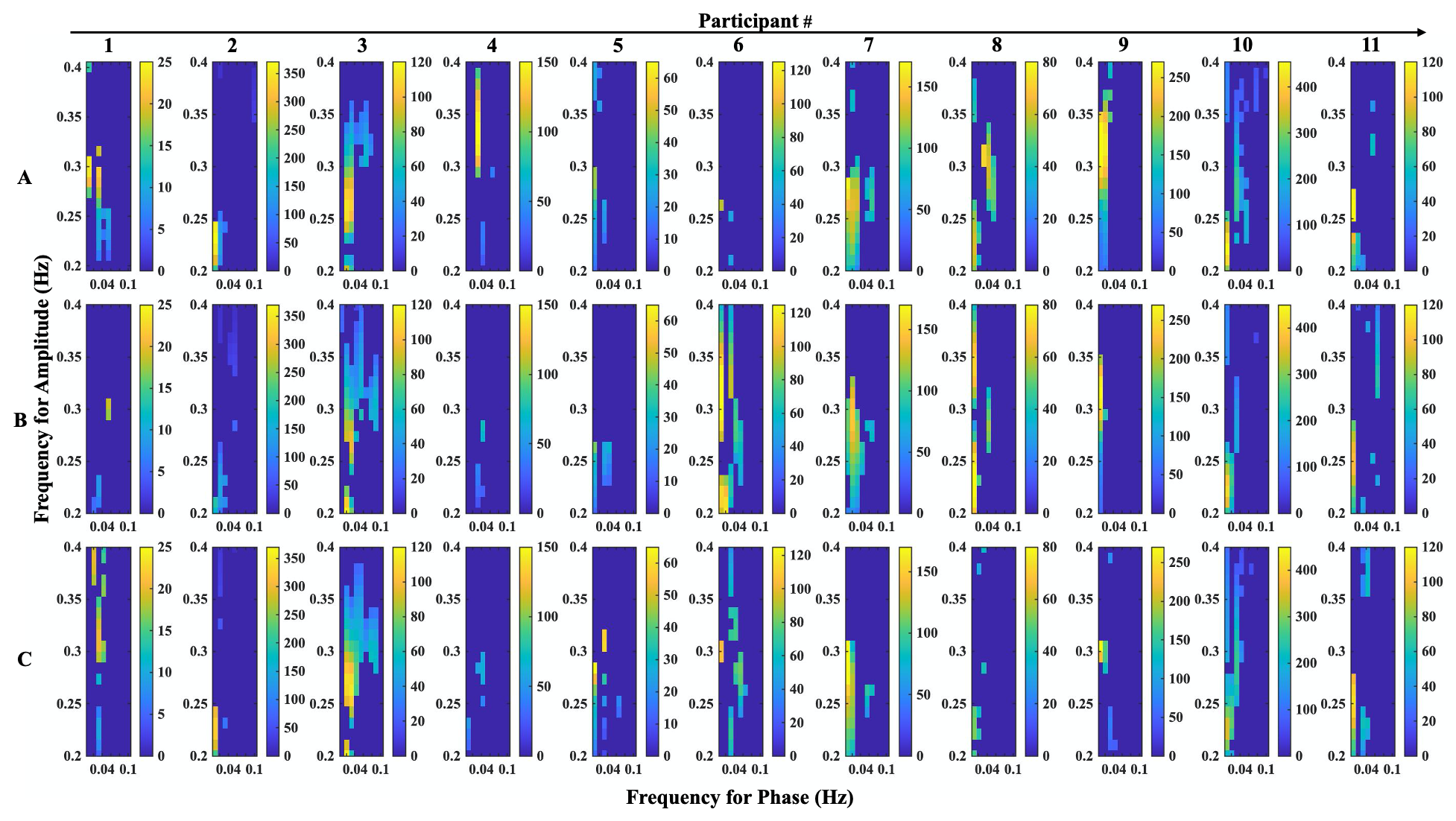
Figure S6: Cross-frequency coupling between respiration and LFOs. Results of coupling quantified by modulation index, between amplitude of RO_Chest_ signal and the phase of (A) LFO_CSF_, (B) LFO_IJV_ and (C) LFO_ICA_ signals for all 11 participants enrolled in the study. Colorbars represent modulation index. ICA – Internal Carotid Artery; IJV – Internal Jugular Vein; CSF - Cerebrospinal Fluid; LFO – Low frequency Oscillations (0.01 Hz – 0.1 Hz); RO – Respiratory Oscillations (0.2 Hz – 0.4 Hz).


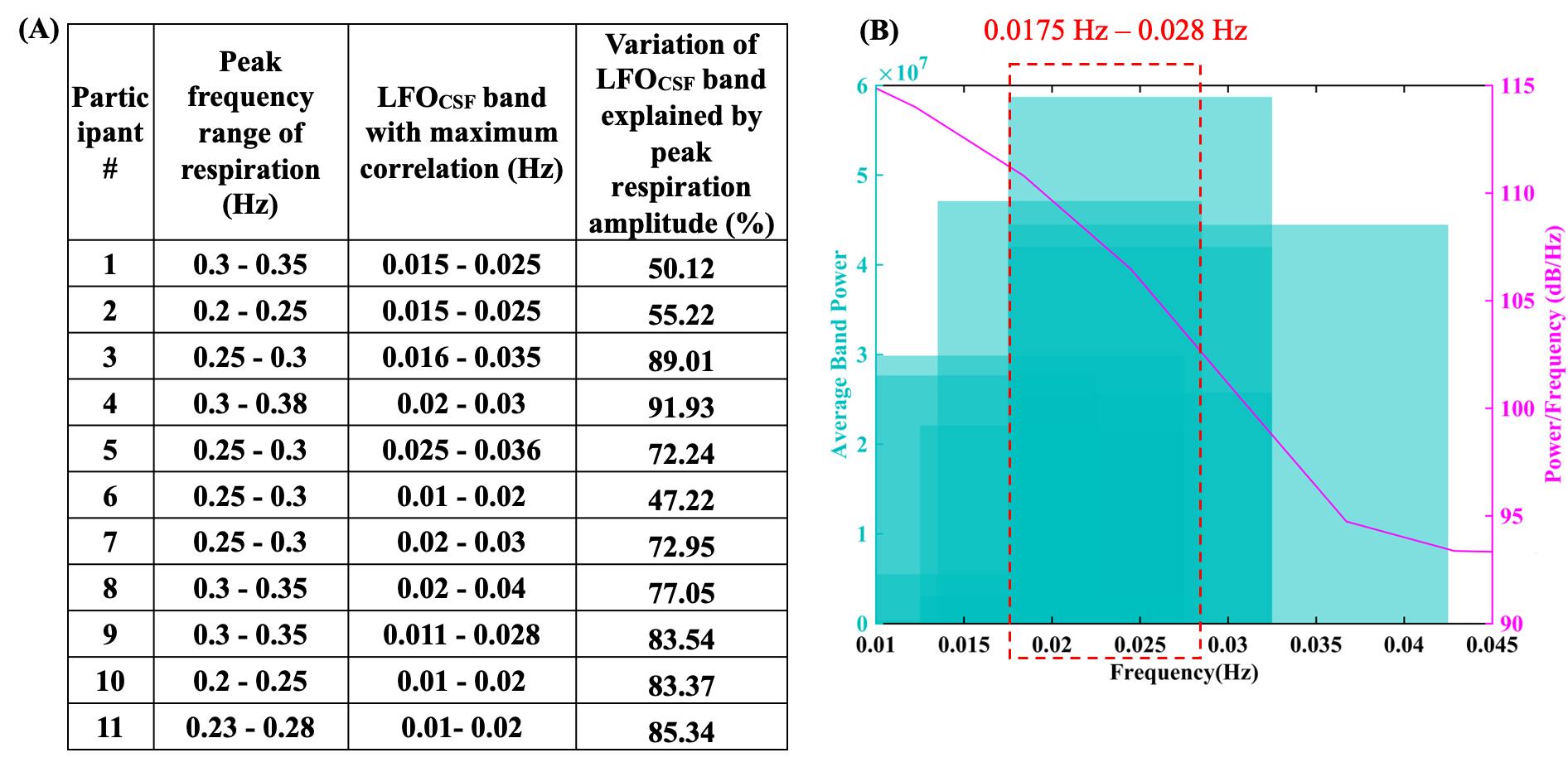


Figure S7: Variation in caudally directed LFO_CSF_ at the fourth ventricle explained by respiration. Specific narrow frequency ranges in LFO_CSF_ with maximum coupling with corresponding peak frequency ranges of respiration was identified for each participant and the percent variability of these narrow LFO_CSF_ bands explained by corresponding peak respiratory frequencies were calculated.(A) Table shows the peak respiratory frequencies and LFO_CSF_ frequencies with maximum correlation for each participant and the corresponding percent variation of peak respiration frequencies in the specific LFO_CSF_ frequency bands. (B) Bar plots of the amplitudes of the average power in the specific LFO_CSF_ frequency bands with maximum correlation with peak respiration show that these LFO_CSF_ bands of all participants overlap in the narrow range of 0.0175 Hz – 0.028 Hz. On average, respiration was found to explain 73.46±15.83% of variability in this narrow LFO range of 0.0175 Hz – 0.028 Hz. The amplitude of average power in each specific LFO_CSF_ frequency band was calculated using the MATLAB function ‘bandpower’. The purple-colored plot shows the average power spectral density curve for all participants. The cyan colored bar plots illustrate the amplitude of average power in the specific LFO_CSF_ bands identified to maximum correlation with their corresponding peak respiratory frequencies. The red highlighted part shows the narrow LFO range with maximum overlap across the participants. CSF - Cerebrospinal Fluid; LFO – Low frequency Oscillations.


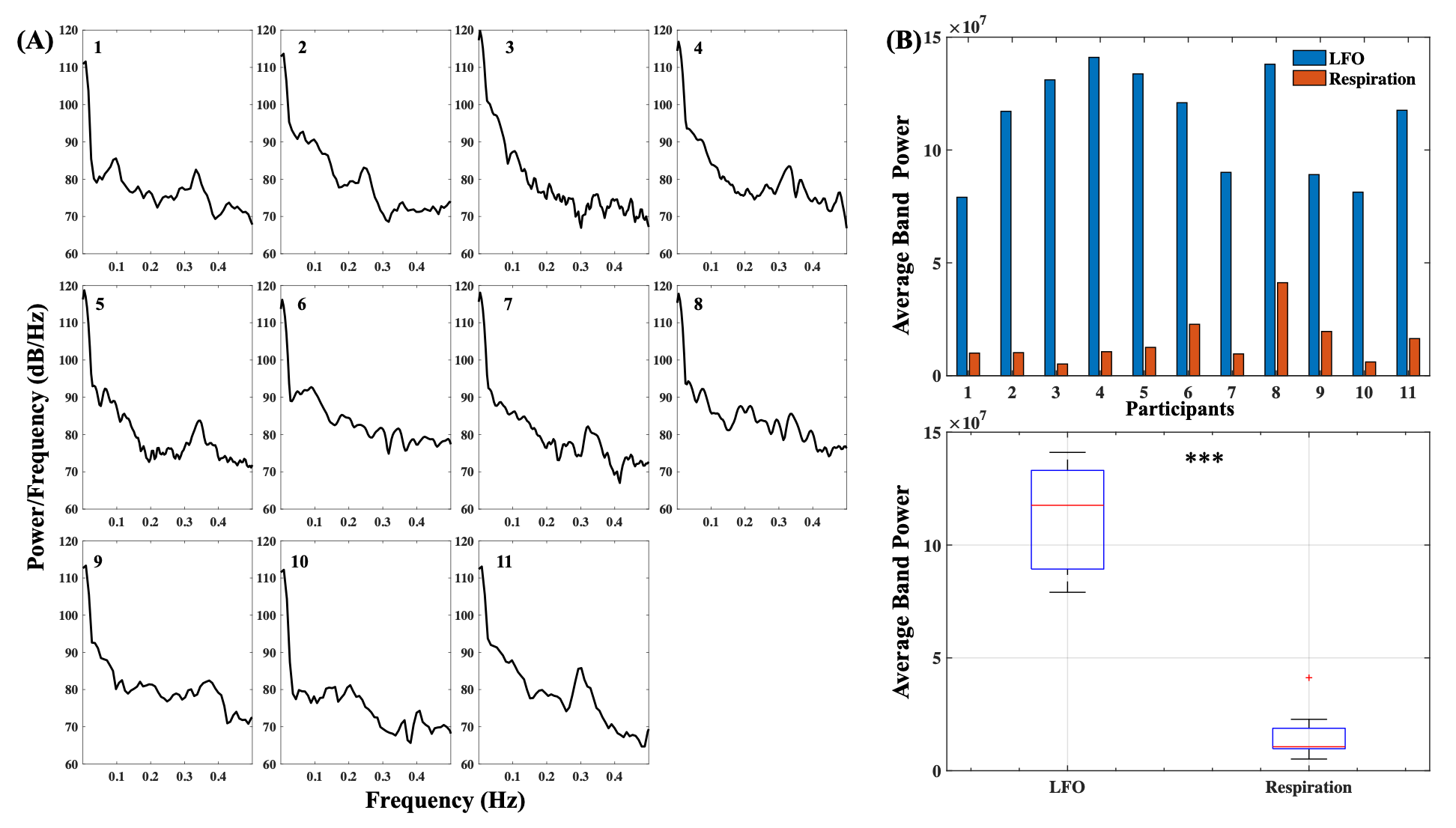


Figure S8: Power analysis of caudally directed CSF signals. (A) Power spectrum of CSF signals of all participants enrolled in the study, clearly illustrates the significant contribution of LFOs, compared to respiration. (B) Average power (amplitude in linear units) of LFO band (0.01 Hz – 0.1 Hz) and respiration band (0.2 Hz – 0.4 Hz) in CSF signals for all participants illustrate that the LFO band power is significantly higher than the respiration band power (p<0.001). The amplitude of average power in each frequency band was calculated using the MATLAB function ‘bandpower’. In the box plot, the red solid line represents the median, box represents the interquartile range, whiskers represent the extreme data points and outliers are represented by the ‘+’ symbol. CSF - Cerebrospinal Fluid; LFO – Low frequency Oscillations (0.01 Hz – 0.1 Hz).

Note1: Low-frequency hemodynamic oscillations in arteries and veins

We would like to mention that the low-frequency hemodynamic oscillations that is of interest in the current study (i.e., hemodynamic LFOs) are in a lower frequency range (0.01 Hz – 0.1 Hz) than the pulsatile volume changes in the arteries brought about by the cardiac cycle. Several previous studies have shown that these low-frequency blood vessel volume oscillations occur in cerebral arteries throughout the brain(1–3). Prior resting state fMRI studies have also detected these oscillations occurring first in the ICAs before they appear in the brain and later on in the IJVs(4,5). In addition, a recent rodent study also illustrated that these vasomotor oscillations in the arteries trigger similar oscillations in the velocity of red blood cells and are thus further conveyed to the draining venules as delayed passive dilations(6). Taken together, it can be gathered that these vessel volume oscillations travel from the ICAs through the cerebral arteries and further induce proportionate blood flow changes in the draining capillaries, venules and veins (i.e., IJV). However, the blood flow changes in IJVs found in our study as well as in other fMRI studies were captured by the deoxyhemoglobin concentration changes (i.e., BOLD effect) caused by these upstream arterial blood volume changes.

Note2: Effects of cardiac pulsation of caudad CSF movement at the level of 4^th^ ventricle

Cardiac pulsations were the first physiological mechanism to be considered as the potential motive force behind CSF flow, in the context of the classic Monro-Kellie Hypothesis. Observations of bidirectional pulsatile CSF flow dependent on the cardiac cycle have been reported using conventional cardiac gated phase contrast MRI based measurements(7–10). Briefly, these studies reported caudally directed displacement of CSF from the brain into the spinal canal to compensate for the increase in intracranial blood volume during cardiac systole and a cranially directed displacement of CSF into the brain from the spinal canal to compensate for the decrease in intracranial blood volume during diastole. However, it is to be noted that these assessments were gated to the cardiac cycle, i.e., CSF flow values quantified were averaged over multiple cardiac cycles. For this reason, the CSF flow related to cardiac pulsations were the only focus and effects of no frequency other than the cardiac frequency on CSF flow were exposed.

The figure S9 illustrates the example CSF (Panel A) and IJV raw traces (Panel B), CSF (Panel C) and IJV (Panel D) pulsations and the CSF (Panel E) and IJV (Panel F) power spectrum from a participant where cardiac pulsations were fully sampled (MRI TR = 0.44 sec). ) Panel G also illustrates the power spectrum of the cardiac waveform extracted from the fMRI scan of the same participant using a software called ‘Happy’(11), which confirms the peak cardiac frequency. Panel A shows that the large amplitude CSF movement peaks in the caudal direction occurs in the low-frequency range. In fact, the cardiac pulsation related movement (>0.6 Hz CSF pulsations illustrated in Panel C) appears to ride on top of these large low-frequency CSF movement. These pulsations however are not capable of changing the direction of these large low-frequency CSF movement peaks. Moreover, power spectrum of the CSF signal (Panel E), clearly show that the cardiac pulsations (~0.88 Hz) only have a negligible effect on CSF movement at the level of 4^th^ ventricle. On average, across all subjects with fully sampled cardiac pulsations (n = 5), these pulsations explained only an 11 percent of total power in the caudally directed CSF signals observed at the fourth ventricle. This therefore shows that heart beat frequency pulsations only exert a minimal influence on CSF movement at the level of 4^th^ ventricle.

Additionally, we ran a simulation to examine the impact of high frequency cardiac signals on the low-frequency 4^th^ ventricle CSF movement. At a sampling frequency of 4 Hz, a hundred high frequency (1-2Hz) signals, each with an amplitude of one third of the 4^th^ ventricle CSF LFO signal (amplitude ratio obtained from one of the participants with fully sampled heart rate with a TR of 440 ms), were generated. This high frequency signal was then mixed with the CSF LFO signal and resampled at 1.04 Hz (equivalent to a TR of 960 ms used in 6 out of 11 participants in our study). The combined signal was then correlated with the original 4^th^ ventricle CSF LFO signal. Panel H in figure S9 illustrates that these correlations remain high above 0.97, indicating that the high frequency cardiac signals could only explain negligible variance in the low frequency CSF movement observed at 4^th^ ventricle. These results are in agreement with a prior study which found that cardiac pulsation-derived LFOs explained only negligible (around 3%) variance in the fMRI data(12).


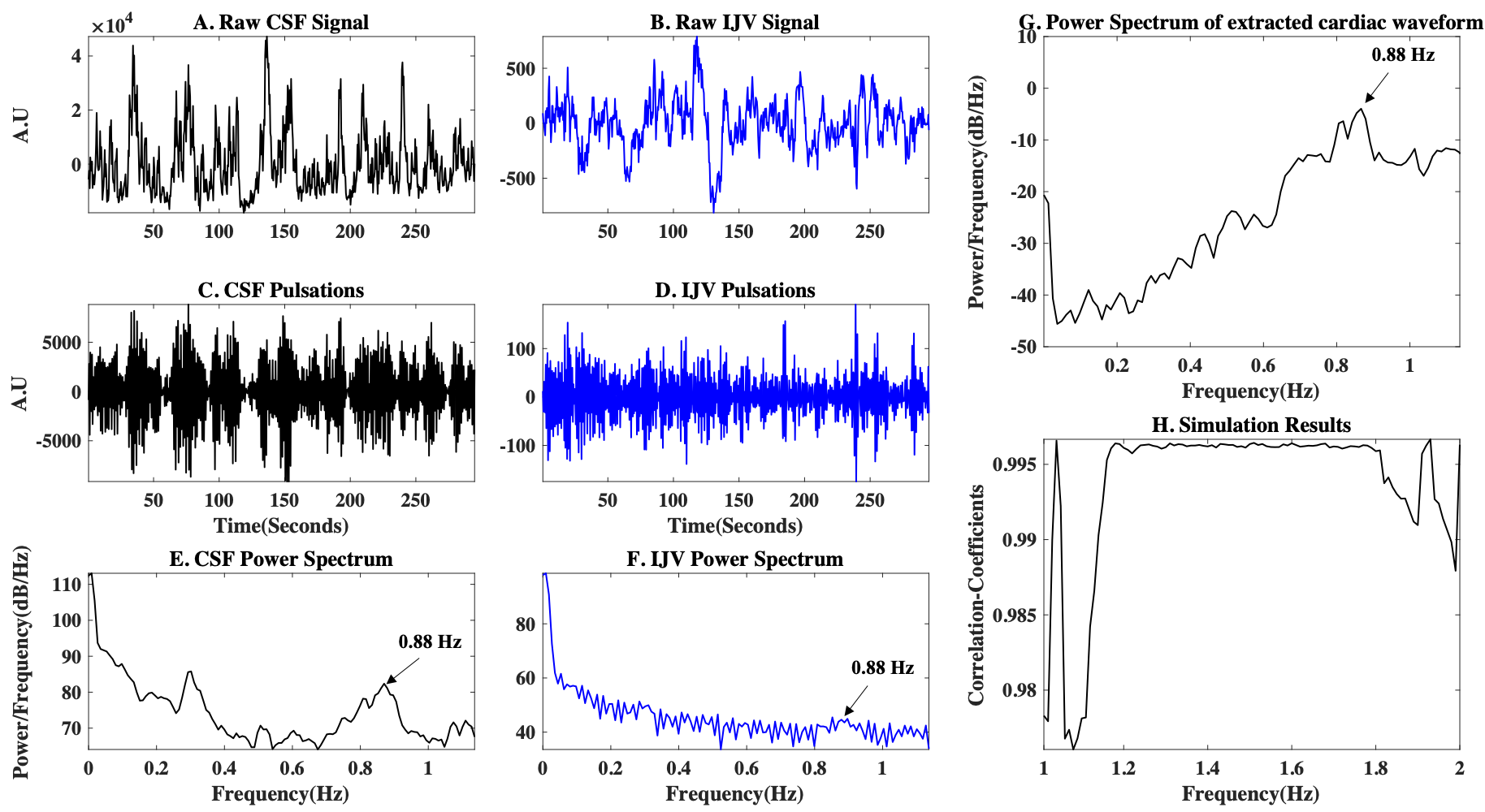


Figure S9: Effect of cardiac pulsations on caudally directed CSF movement. Example raw (A) CSF and (B) IJV traces, (C) CSF and (D) IJV pulsations (>0.6 Hz), the (E) CSF and (F) IJV power spectrum estimates and (G) power spectrum of the extracted cardiac waveform from a participant where cardiac pulsations were fully sampled (MRI TR = 0.44 sec). Panel H shows the simulation results of the effect of high frequency cardiac signals on low-frequency 4^th^ ventricle caudad CSF movement. A.U – Arbitrary Units; IJV – Internal Jugular Vein; CSF - Cerebrospinal Fluid.

**References:**

1. Tong Y, Frederick BD. Time lag dependent multimodal processing of concurrent fMRI and near-infrared spectroscopy (NIRS) data suggests a global circulatory origin for low-frequency oscillation signals in human brain. Neuroimage. 2010 Nov;53(2):553–64.

2. Osol G, Halpern W. Spontaneous vasomotion in pressurized cerebral arteries from genetically hypertensive rats. Am J Physiol. 1988 Jan;254(1 Pt 2):H28-33.

3. Drew PJ. Vascular and neural basis of the BOLD signal. Curr Opin Neurobiol. 2019 Oct;58:61–9.

4. Tong Y, Yao J (Fiona), Chen JJ, Frederick B de B. The resting-state fMRI arterial signal predicts differential blood transit time through the brain. J Cereb Blood Flow Metab. 2018;39(6):1148–60.

5. Yao J, Wang JH, Yang HC, Liang Z, Cohen-Gadol AA, Rayz VL, et al. Cerebral circulation time derived from fMRI signals in large blood vessels. J Magn Reson Imaging. 2019;50(5):1504–13.

6. Rungta RL, Chaigneau E, Osmanski B-F, Charpak S. Vascular Compartmentalization of Functional Hyperemia from the Synapse to the Pia. Neuron. 2018 Jul;99(2):362-375.e4.

7. Bhadelia RA, Bogdan AR, Wolpert SM. Analysis of cerebrospinal fluid flow waveforms with gated phase-contrast MR velocity measurements. AJNR Am J Neuroradiol. 1995 Feb;16(2):389–400.

8. Enzmann DR, Pelc NJ. Cerebrospinal fluid flow measured by phase-contrast cine MR. AJNR Am J Neuroradiol. 1993;14(6):1301–10.

9. Greitz D, Wirestam R, Franck A, Nordell B, Thomsen C, Ståhlberg F. Pulsatile brain movement and associated hydrodynamics studied by magnetic resonance phase imaging. The Monro-Kellie doctrine revisited. Neuroradiology. 1992;34(5):370–80.

10. Mestre H, Tithof J, Du T, Song W, Peng W, Sweeney AM, et al. Flow of cerebrospinal fluid is driven by arterial pulsations and is reduced in hypertension. Nat Commun [Internet]. 2018;9(1). Available from: http://dx.doi.org/10.1038/s41467-018-07318-3

11. Aslan S, Hocke L, Schwarz N, Frederick B. Extraction of the cardiac waveform from simultaneous multislice fMRI data using slice sorted averaging and a deep learning reconstruction filter. Neuroimage [Internet]. 2019;198(April):303–16. Available from: https://doi.org/10.1016/j.neuroimage.2019.05.049

12. Hocke LM, Tong Y, Lindsey KP, de B Frederick B. Comparison of peripheral near-infrared spectroscopy low-frequency oscillations to other denoising methods in resting state functional MRI with ultrahigh temporal resolution. Magn Reson Med. 2016 Dec;76(6):1697–707.
